# Neuroimaging Correlates of the NIH-Toolbox-Driven Cognitive Metrics in Children

**DOI:** 10.31083/j.jin2312217

**Published:** 2024-12-12

**Authors:** Hector Acosta-Rodriguez, Cuiping Yuan, Pratheek Bobba, Alicia Stephan, Tal Zeevi, Ajay Malhotra, Anh Tuan Tran, Simone Kaltenhauser, Seyedmehdi Payabvash

**Affiliations:** 1 Department of Radiology and Biomedical Imaging, Yale School of Medicine, New Haven, CT 06519, USA; 2 Institute of Diagnostic and Interventional Radiology, University Hospital Zurich, 8091 Zurich, Switzerland

**Keywords:** NIH toolbox, fluid intelligence, crystalized cognition, diffusion tensor imaging, functional MRI

## Abstract

**Background:**

The National Institutes of Health (NIH) Toolbox Cognition Battery is increasingly being used as a standardized test to examine cognitive functioning in multicentric studies. This study examines the associations between the NIH Toolbox Cognition Battery composite scores with neuroimaging metrics using data from the Adolescent Brain Cognitive Development (ABCD) study to elucidate the neurobiological and neuroanatomical correlates of these cognitive scores.

**Methods:**

Neuroimaging data from 5290 children (mean age 9.9 years) were analyzed, assessing the correlation of the composite scores with Diffusion Tensor Imaging (DTI), structural Magnetic Resonance Imaging (sMRI), and resting-state functional connectivity (rs-fMRI). Results were adjusted for age, sex, race/ethnicity, head size, body mass index (BMI), and parental income and education.

**Results:**

Higher fluid cognition composite scores were linked to greater white matter (WM) microstructural integrity, lower cortical thickness, greater cortical surface area, and mixed associations with rs-fMRI. Conversely, crystallized cognition composite scores showed more complex associations, suggesting that higher scores correlated with lower WM microstructure integrity. Total cognition scores reflected patterns consistent with a combination of both fluid and crystallized cognition, but with diluted specific insights. Our findings highlight the complexity of the neuroimaging correlates of the NIH Toolbox composite scores.

**Conclusions:**

The results suggest that fluid cognition composite scores may serve as a marker for cognitive functioning, emphasizing neuroimaging’s clinical relevance in assessing cognitive performance in children. These insights can guide early interventions and personalized education strategies. Future ABCD follow-ups will further illuminate these associations into adolescence and adulthood.

## 1. Introduction

The National Institutes of Health (NIH) Toolbox for the Assessment of Neurological and Behavioral Function is a set of brief computerized measures designed to assess sensory, motor, emotional, and cognitive functioning [1–3]. It was developed by the NIH Blueprint for Neuroscience Research, as a collaborative effort among 16 NIH Institutes [4]. The NIH Toolbox was created to address several issues in current research practices. Existing measures used to assess neurological and behavioral function often lack uniformity, are expensive, tested on non-diverse populations, difficult to administer, and not suitable for all age groups [1]. These inconsistencies make it challenging to compare and pool data across different studies, which is necessary for obtaining large and diverse samples. The NIH Toolbox aims to provide brief assessment tools, which are inclusive, and cover various age ranges and demographic groups, with versions available in English and Spanish [1]. While not intended to replace in-depth assessments or serve as diagnostic tools, the NIH Toolbox enhances data collection in large cohort studies and advances biomedical research by providing a common metric for assessing neurological and behavioral function across diverse study designs and populations.

Among the four batteries established by the NIH Toolbox, the NIH Toolbox Cognitive Battery (NIHTB-CB) has been the most widely used by researchers and clinicians [5]. The NIHTB-CB provides three age-corrected T-scores, which compare the test-taker’s performance to a nationally representative normative sample from the NIH Toolbox, adjusting for key demographic variables collected during the national norming study [6]. The three scores obtained are the NIH Toolbox Fluid Cognition Composite Score (NIHTB-FCCS), the NIH Toolbox Crystallized Cognition Composite Score (NIHTB-CCCS), and the NIH Toolbox Cognitive Function Composite Score (NIHTB-CFCS). These scores are derived from the Cattell–Horn–Carroll component theory of cognitive abilities, which establishes intelligence as a dynamic interaction between different intelligences such as fluid and crystallized, developing and transforming throughout the life span [7,8]. Fluid abilities are used to solve problems, think and act quickly, and encode new episodic memories. These abilities are presumed to be especially influenced by biological processes and less dependent on past exposure (learning experiences). They tend to be more sensitive to neurobiological integrity, including changes in brain functioning with aging and in a variety of neurological disorders that alter brain structure and function [9,10]. Crystallized abilities, in contrast, are presumed to be more dependent on experience, and less by biological influences. They represent accumulated store of verbal knowledge and skills [9,10].

These composite scores allow for a general interpretation and evaluation of overall cognitive functioning, offering a higher level of reliability than any individual test alone. These scores obtained from the NIHTB-CB have recently gained popularity as a standardized method for assessing cognitive performance. Recent studies have started to specifically utilize the cognitive battery composite scores to analyze the age related neurodevelopmental changes in children [11,12] and the cognitive function of individual with intellectual disabilities [13]. Analyzing the correlation of the NIHTB-CB composite scores with well stablished neuroimaging metrics such as Diffuse Tensor Imaging, structural Magnetic Resonance Imaging (MRI), and resting-state functional connectivity MRI, which have been proved instrumental to test children cognitive function, will provide important information as to the validity of these scores for measuring child neurodevelopment.

Diffusion Tensor Imaging (DTI) is an MRI technique that measures the anisotropic diffusion of water molecules in brain tissue, primarily along axonal fibers in white matter (WM), providing crucial insights into brain cellular architecture and connectivity. These parameters are vital for assessing the integrity and connectivity of brain networks, particularly in the context of cognition and neurodevelopment [14–16]. By modeling these diffusion patterns in three dimensions, DTI allows for the computation of various diffusion characteristics, such as fractional anisotropy (FA), mean diffusivity (MD), axial diffusivity (AD), and radial diffusivity (RD) [17,18]. Additionally, multi-compartmental modeling from multi-shell diffusion MRI provides restriction spectrum metrics such as neurite density (ND). FA measures the degree of directionality of water diffusion, higher in well-organized WM tracts. ND quantifies the intra-cellular volume fraction, indicating the proportion of brain parenchymal volume occupied by neurites (axons and dendrites) [19]. AD measures water diffusion along the principal axis of WM tracts, typically aligned with the axons, while RD measures water diffusion perpendicular to this axis. MD represents the average rate of water diffusion in all directions.

Structural MRI (sMRI) provides detailed images of the brain’s structure. It measures cortical thickness, the distance between the WM and the brain’s surface, and surface area, the total area of the cortex. These metrics are essential for understanding children brain neurocognitive development [20–22]. Resting-state functional MRI (rs-fMRI) measures spontaneous fluctuations in blood oxygen level-dependent signals across the brain. It identifies functional connectivity between different brain regions. This technique helps to understand brain networks and their alterations in brain development [23–25]. Previous studies have analyzed the neuroimaging correlates of other pre-established objective measures of cognitive scores. For example, the Stanford-Binet Intelligence Test-4 has been previously used to analyze the WM of typically developing and dyslexic children [26] and the Kaufman Assessment Battery for Children has been used to study children’s brain functional networks [27]. However, although studies have independently validated the use of the NIHTB-CB in relation to child neurocognitive development [28] and others have examined the impact of neurocognition on neuroimaging metrics [29], there is limited research exploring the relationship between NIHTB-CB composite scores and neuroimaging metrics. Investigating these correlates could significantly enhance our understanding of the validity and utility of NIHTB-CB scores in assessing children cognitive development, as previous studies have already related the importance of neuroimaging metrics on studying cognition [30] and intellectual disabilities [31].

In our study, we utilized the Adolescent Brain Cognitive Development (ABCD) dataset, a rich and nationally representative dataset, to examine the correlations of the NIHTB-CB composite scores on children neurocognition. This dataset is uniquely comprehensive, encompassing a cohort of over 11,800 children aged 9–10, at baseline, from 21 diverse sites across the United States, ensuring a broad representation of socio-demographic backgrounds and neighborhood environments [32,33]. Our analysis centered on exploring how the different NIHTB-CB composite scores relate to the different well established neuroimaging metrics in children. The goal was to determine how well the different composite scores correlated to the patterns of neurocognitive development previously observed in children utilizing neuroimaging metrics. This study underscores the clinical relevance of using neuroimaging to identify cognitive performance in children based on widely used NIH Toolbox metrics. These insights can guide early therapeutic interventions and personalized educational strategies. Additionally, clinical fields have been increasingly adopting a variety of assessment tools, combining cognitive and neurobiological measures to enhance understanding of patients [34]. Thus, this study analyzing the effectiveness of the NIH Toolbox Cognitive Battery in children could also prove to be useful in clinical medicine.

## 2. Methods

### 2.1 ABCD Database

In this study we retrospectively analyzed the demographic, cognition, and neuroimaging data of 11,868 subjects in the ABCD study (5.1) (https://abcdstudy.org/). The data for cognition, demographic and neuroimaging data was assessed at the first visit. Subjects were between 9 and 10 years old at the time of enrollment from September 2016 to August 2018, and they were gathered from 21 different research centers, all of which had received the necessary approval from institutional review boards (IRBs), including centralized approval from the University of California, San Diego. The study was carried out in accordance with the guidelines of the Declaration of Helsinki. Consent from the participants’ parents and the children’s consent were duly obtained [35]. The ABCD study excluded any subjects who had significant sensory, neurological, medical, or intellectual impairments, those who could not undergo an initial MRI scan, or those who were not proficient in English [36,37].

### 2.2 Inclusion of Participants

In this study, we selected participants from the ABCD study who had complete data, which included demographic details (such as age, gender, weight, height, handedness, race, parents’ education level, and parental income), neuroimaging data (including diffusion tensor imaging, resting-state functional MRI, and cortex morphology), and complete NIH Toolbox cognition data. Individuals with any neuroimaging signs of pathology, a past traumatic brain injury, or any psychiatric or developmental conditions as determined by the Kiddie Schedule for Affective Disorders and Schizophrenia for the Diagnostic and Statistical Manual of Mental Disorders, Fifth Edition-5 (KSADS-5) were excluded from this study [38]. In the same way, those subjects with extreme body mass index (BMI) z-scores (below −4 or above 8) were also excluded [39].

### 2.3 Anthropometric and Sociodemographic Data

The growthcleanr package within the R programming language [40] was used to calculate the BMI z-scores for the subjects, using age- and sex-specific values provided by WHO. The race of the subjects was categorized into five main groups: White, Black, Asian, Hispanic, and multiracial or other categories. The highest education level for either parent of the subjects was documented using a spectrum of 21 distinct levels, which ranged all the way from primary schools to postgraduate degrees. Combined parental income for all subjects was captured in 10 distinct ranges, ranging from less than $5000 to over $200,000.

### 2.4 Cognition Data

The NIH Toolbox Cognition Battery is a brief and comprehensive assessment utilized to determine the cognitive functioning of an individual [41]. This battery was utilized to evaluate cognitive performance in our study. The battery includes a series of tasks administered via iPad, designed to measure various cognitive domains. For our study, we focused on the composite scores derived from these tasks, specifically the Fluid Cognition Composite Score, Crystallized Cognition Composite Score, and Cognitive Function Composite Score. The Fluid Cognition Composite Score includes five tests designed to measure attention, cognitive control, executive function, episodic memory, and processing speed. These tests are the Flanker Inhibitory Control & Attention Test, the Picture Sequence Memory Test, the Dimensional Change Card Sort Test, the Pattern Comparison Processing Speed Test, and the List Sorting Working Memory Test. The Crystallized Cognition Composite Score includes tests that assess language, vocabulary knowledge and oral reading skills. These tests are the Picture Vocabulary Test and the Oral Reading Recognition Test. Finally, the Cognitive Function Composite Score integrates both the Fluid and Crystallized scores (all seven tests) and aims to provide a comprehensive measure of cognitive performance. All seven tests of the NIH Toolbox were administered, allowing for the calculation of all composite scores.

### 2.5 Neuroimaging Metrics Data

Harmonized protocols were implemented across different 3-Tesla MRI systems (Siemens, DISCOVERY MR750, Munich, Germany; General Electric, Achieva dStream or Ingenia, Boston, MA, USA; Philips, Prisma, Amsterdam, Netherlands.) at all participating locations to acquire structural, diffusion, and functional MRI. Using guidelines suggested by the ABCD study, any subjects with pathological findings which required any type of clinical referrals, as determined by a certified neuroradiologist [42], or those who scans did not pass the quality control or the FreeSurfer cortical surface reconstruction review were eliminated from the study. Corrections were applied to all images to adjust for any distortions and movements.

Structural MRI data was derived by segmenting the cortical surface of T1-weighted scans and aligning them with a surface-based atlas through nonlinear registration using FreeSurfer software, version 5.3.0, Laboratories for Computational Neuroimaging, Boston, Massachusetts, United States [42,43]. Cortical thickness, surface areas, and sulcal depths across 68 cortical regions were identified according to the Desikan-Killiany atlas, and the intercranial volumes was measured with the Automatic Segmentation (ASEG) atlas [44].

Diffusion MRI imaging was obtained by using multi b-value, multi-directional sequences. The processing for the diffusion MRI images included correction for head motion and rotation, correction for gradient nonlinearity distortion, eddy current correction, and B0 distortion correction. All the T2-weighted B0 images which were obtained were then registered to a series of T1 weighted structural images. This new T1 weighted structural images where then transferred to a standard 1.7 mm isotropic space. DTI and restriction spectrum imaging (RSI) metrics, such as fractional anisotropy (FA), mean diffusivity (MD), radial diffusivity (RD), axial diffusivity (AD), and neurite density (ND) were obtained. DTI and RSI metrics across 35 WM tracts were segmented and measured using AtlasTrack [36,45].

Resting state functional MRI (rs-fMRI) data was gathered over four successive 5-minute runs, all of which added to a total of a 20-minute session. The fMRI datasets underwent a standardization protocol using the Multi-Modal Processing Stream package. This standardization included corrections for head motion, corrections for B0 field distortions, corrections for gradient nonlinearity distortion, inter-scan movements corrections, followed by the co-registration of T2-weighted functional scans to their T1-weighted structural scans. Further processing of the imaging data includes the elimination of quadratic trends and time courses attributable to WM, cerebral ventricles, and the whole brain by using linear regressions. Subsequently, motion regression was applied to eliminate frames exceeding a threshold of 0.3 mm. Time courses filtering was employed to isolate and include only those between 0.009 and 0.08 Hz. Any additional head or respiratory motion were removed using motion censoring. Gordon parcel atlas was used to segment and label functional connectivity patterns. Within and between-network correlations were then calculated across the different pairs of functional networks, resulting in 91 functional connectivity correlations of interest. These regions of interest (ROIs) are easily replicable and freely available within the data release 5.1.

### 2.6 Statistical Analysis

The relationship between the different NIH Toolbox composite scores and neuroimaging metrics was evaluated. We applied mixed linear models to determine the association between the NIH Toolbox composite scores with average DTI metrics of each WM tract, cortex morphology, and fMRI metrics, using Python’s “statsmodels” library. For DTI, the average metrics of each WM tract were the dependent variables. For cortex morphology, the dependent variables were cortical thickness and cortical surface area of brain regions. For fMRI, the dependent variables were the connectivities of functional brain networks. In all models, the NIH Toolbox composite scores along with demographic and biological covariates were independent predictors. All mixed linear models employed accounted for covariates such as the participant’s age at imaging, gender, BMI z-score at the time of imaging, handedness, race, and cranial volume at imaging time. Additionally, the highest educational level of parents and combined parental income were adjusted for as random effects in the mixed linear model analysis. A *p*-value threshold of 0.05 was considered indicative of significance, and all *p*-values were adjusted for multiple comparisons using the Benjamini-Hochberg method [46]. R was utilized for data cleaning and for structural MRI and functional fMRI data visualization, using packages such as tidyverse [47], dplyr [48], ggplot2 [49], cowplot [50], ggseg [51], and circlize [52]. tidyverse [47], is a collection of R packages that allows tasks such as data manipulation and visualization, providing an efficient way for handling large datasets. dplyr [48], a component of tidyverse, was specifically designed for efficient data manipulation, allowing for the transformation, filtering, and summarization of large datasets. ggplot2 [49] is used to create data visualizations and in our study allowed for the mapping of cortical thickness or surface values to specific color gradients. cowplot [50] helps to combine multiple ggplot2 plots into a single figure, useful for arranging brain plots and legends together for clear presentation. ggseg [51] is specific for neuroimaging data visualization, specifically for plotting brain atlases, and was used to display cortical regions based on the processed neuroimaging data. Lastly, circlize [52] allows the creation of chord diagrams, useful for representing relationships such as functional connectivity between brain regions. Mixed linear model analysis and data visualization for DTI were conducted using Python, with libraries including nilearn, matplotlib, and numpy. nilearn is a widely used Python library in neuroimaging studies for tasks such as image processing and visualization of brain WM microstructure (DTI). matplotlib provides a comprehensive plotting framework for creating high-quality, customizable data visualizations. Lastly, numpy is a fundamental library offering tools for handling and performing complex mathematical operations on large datasets, crucial for efficiently processing the imaging data, demographic data and the statistical outputs.

## 3. Results

### 3.1 Subject Characteristics

**Fig. 1.**
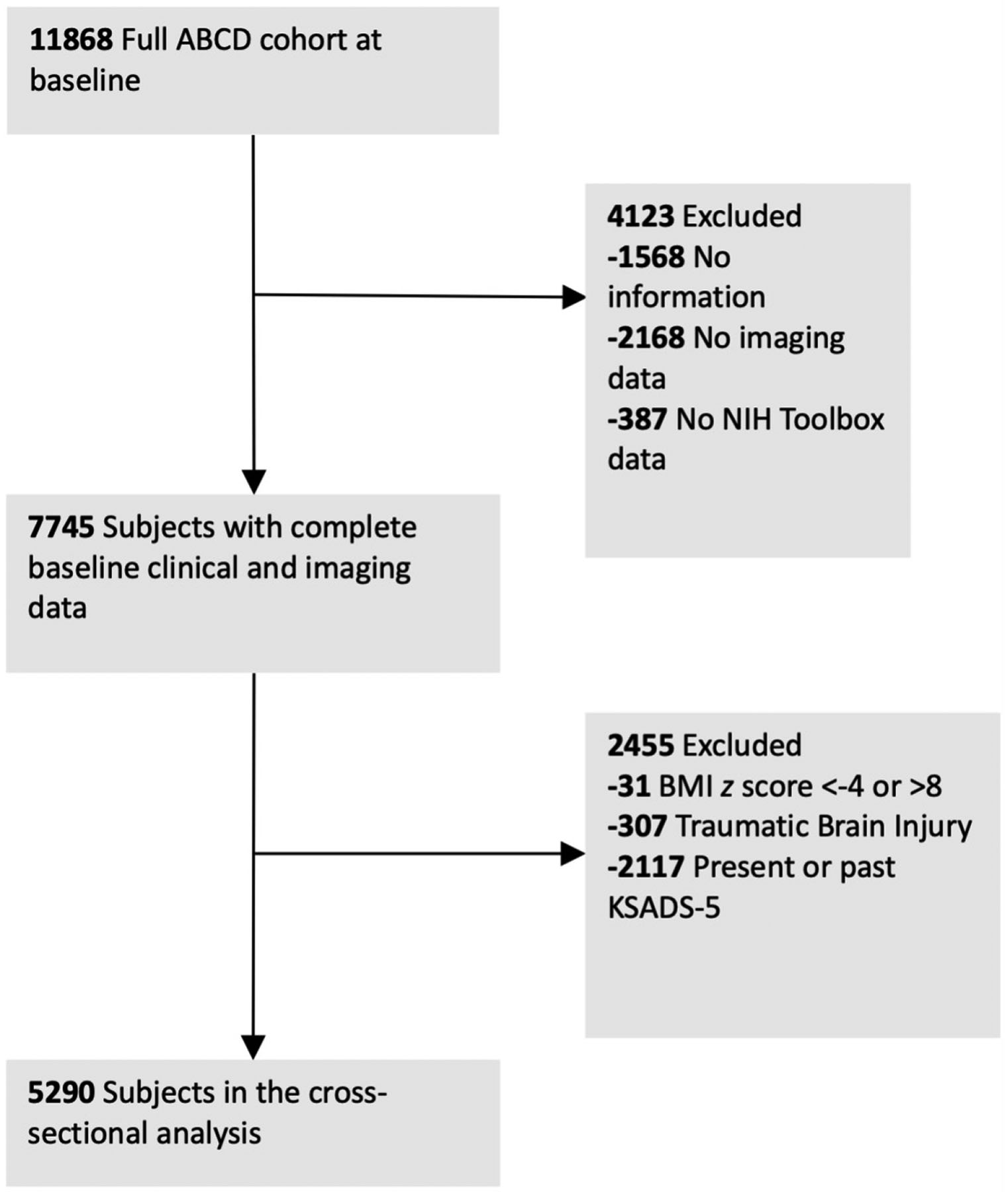
Flowchart depicting the inclusion of subjects for the analysis. ABCD, Adolescent Brain Cognitive Development; NIH, National Institutes of Health; BMI, body mass index; KSADS-5, Kiddie Schedule for Affective Disorders and Schizophrenia for the Diagnostic and Statistical Manual of Mental Disorders, Fifth Edition-5.

Fig. 1 displays the inclusion flowchart of subjects for the analysis from the overall ABCD study dataset. A total of 5290 children, with a mean (standard deviation, SD) age of 9.9 ± (0.6) years, were included in the analysis. Table 1 summarizes the demographic characteristics of subjects included in the study.

### 3.2 Association of NIH Toolbox Cognition Composite Scores with WM Microstructure

**Fig. 2.**
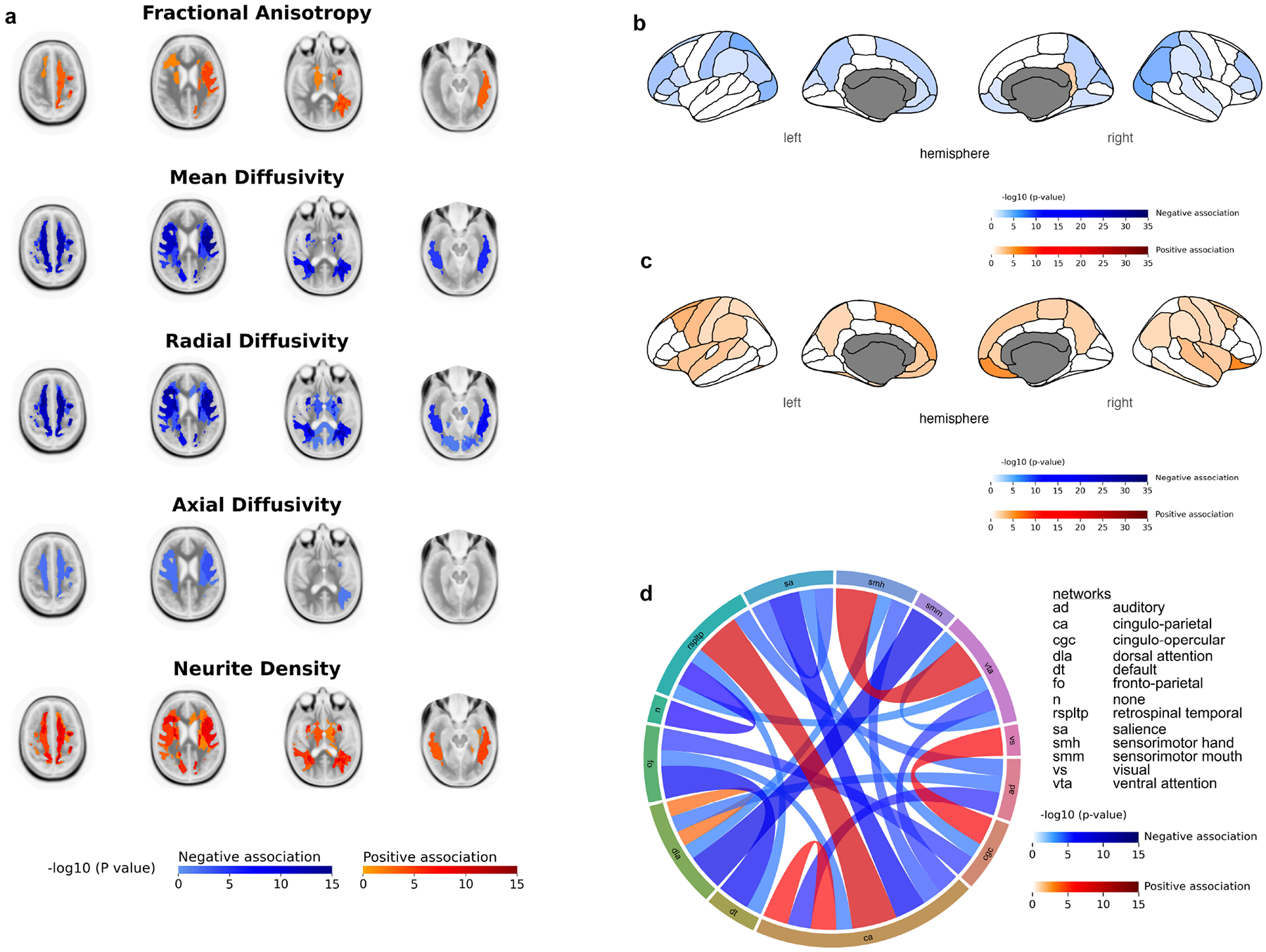
Mixed linear model analyses of the NIH Toolbox Fluid Cognition Composite Score with different neuroimaging metrics. (a) Diffusion tensor imaging metrics. (b) Cortical Thickness. (c) Cortical Surface Area. (d) Association of NIH Toolbox Fluid Cognition Composite Score with functional connectivity between brain networks: the connecting columns’ thickness and color intensity represent the strength of relationship (–log (*p* value)); thus, a thicker intensely red column shows stronger positive association of internetwork connectivity with Fluid Cognition Composite Score. Values shown were corrected for subjects’ age at time of imaging, sex, BMI z-score at time of imaging, handedness, race, and cranial volume at time of imaging as covariates and adjusting for the highest parental level of education and combined parental income level as random effects. Blue areas depict regions of significant (false discovery rate corrected *p*-value < 0.05) negative association while red areas depict regions of significant (false discovery rate corrected *p*-value < 0.05) positive association.

The analysis of WM diffusion metrics revealed that higher scores on the NIH Toolbox Fluid Cognition Composite Score were associated with significantly higher average FA and ND predominantly at the corticostriate projections via the external capsule to superior parietal cortex, the left corticostriate projections via the external capsule to superior frontal cortex (FA: *β* = 9.56 × 10^−4^, *p* = 3.04 × 10^−2^; ND: *β* = 1.03 × 10^−3^; *p* = 2.73 × 10^−3^) and left parietal and temporal superior longitudinal fasciculus WM tracts (Fig. 2a). Conversely, higher fluid cognition scores correlated with significantly lower average MD and RD in these same tracts (Fig. 2a). AD also displayed a negative association with the score, within the corticocortical projections from inferior frontal cortex to superior frontal cortex and the parietal superior longitudinal fasciculus (Fig. 2a). Supplementary Tables 1–5 list the WM tracts where there were significant associations between the NIH Toolbox Fluid Cognition Composite Score and diffusion metrics.

**Fig. 3.**
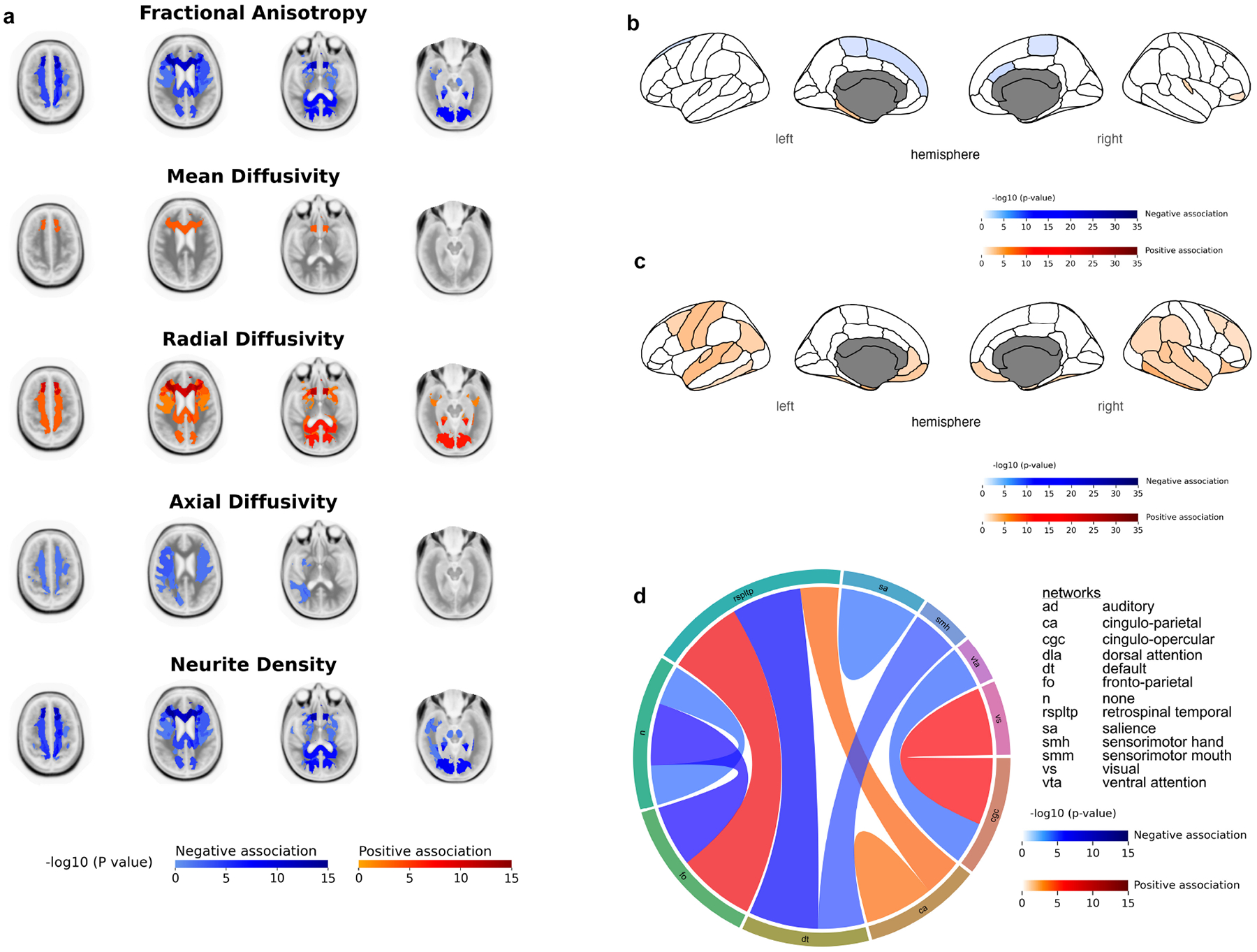
Mixed linear model analyses of the NIH Toolbox Crystallized Cognition Composite Score with different neuroimaging metrics. (a) Diffusion tensor imaging metrics. (b) Cortical Thickness. (c) Cortical Surface Area. (d) Association of NIH Toolbox Crystallized Cognition Composite Score with functional connectivity between brain networks: the connecting columns’ thickness and color intensity represent the strength of relationship (–log (*p* value)); thus, a thicker intensely red column shows stronger positive association of internetwork connectivity with Crystallized Cognition Composite Score. Values shown were corrected for subjects’ age at time of imaging, sex, BMI z-score at time of imaging, handedness, race, and cranial volume at time of imaging as covariates and adjusting for the highest parental level of education and combined parental income level as random effects. Blue areas depict regions of significant (false discovery rate corrected *p*-value < 0.05) negative association while red areas depict regions of significant (false discovery rate corrected *p*-value < 0.05) positive association.

For the NIH Toolbox Crystallized Cognition Composite Score, higher scores were associated with significantly lower average FA and ND values primarily in the forceps minor and major, the corpus callosum (FA: *β* = −1.96 × 10^−3^, *p* = 5.85 × 10^−5^; ND: *β* = −1.49 × 10^−3^, *p* = 8.86 × 10^−5^), the corticostriate projections to inferior frontal cortex, left cingulate cingulum, and right uncinate (Fig. 3a). AD also showed a negative association with the corticocortical projections from inferior frontal cortex to superior frontal cortex, the right corticostriate projections via the external capsule to superior parietal cortex, and the right parietal superior longitudinal fasciculus (Fig. 3a). Conversely, higher scores were associated with higher average MD and RD values, with increased MD observed only at the forceps minor, and increased RD observed mainly at the same tracts as FA and ND (Fig. 3a). Supplementary Tables 6–10 list the WM tracts where there were significant associations between the NIH Toolbox Crystallized Cognition Composite Score and diffusion metrics.

**Fig. 4.**
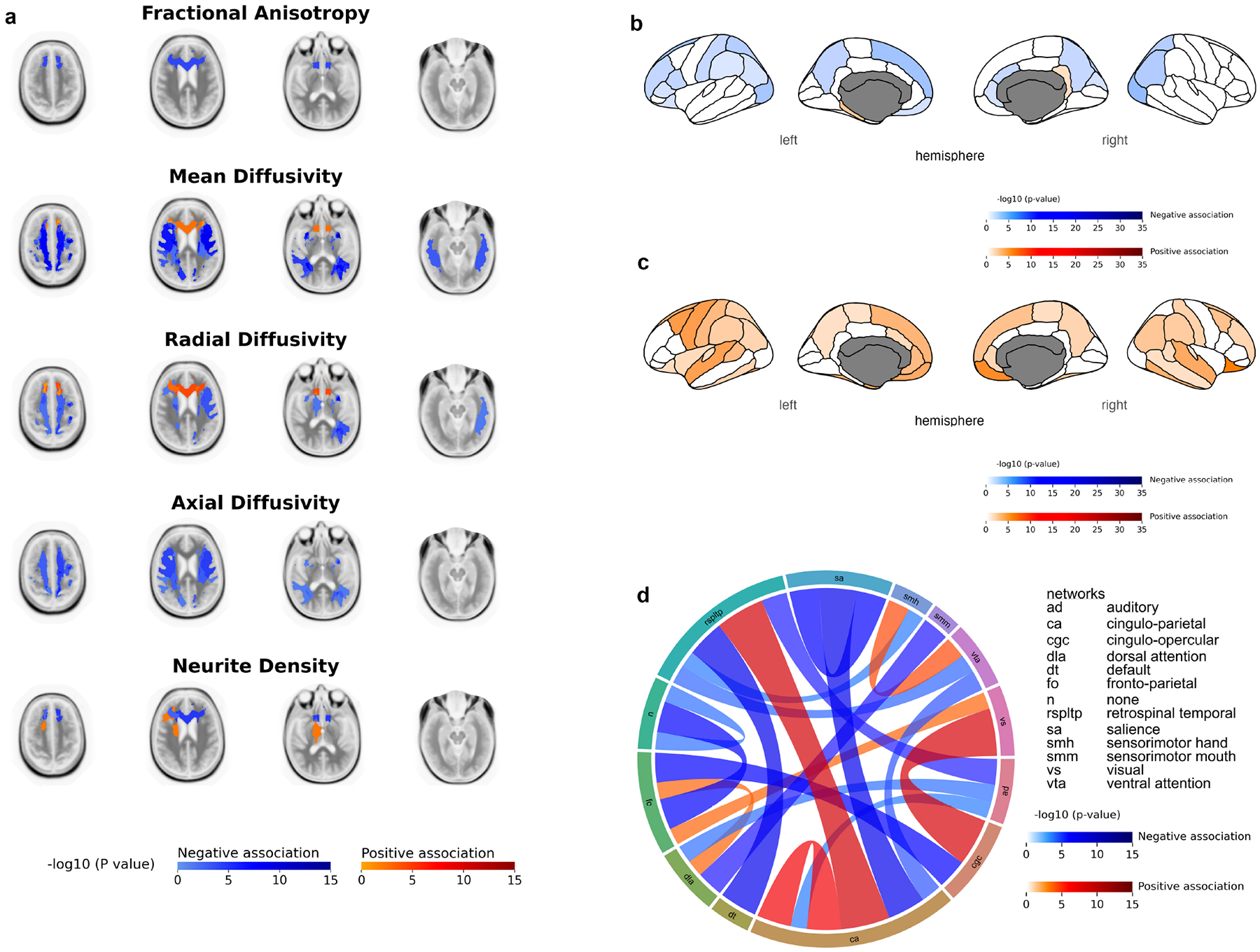
Mixed linear model analyses of the NIH Toolbox Cognitive Function Composite Score with different neuroimaging metrics. (a) Diffusion tensor imaging metrics. (b) Cortical Thickness. (c) Cortical Surface Area. (d) Association of NIH Toolbox Cognitive Function Composite Score with functional connectivity between brain networks: the connecting columns’ thickness and color intensity represent the strength of relationship (–log (*p* value)); thus, a thicker intensely red column shows stronger positive association of internetwork connectivity with Cognitive Function Composite Score. Values shown were corrected for subjects’ age at time of imaging, sex, BMI z-score at time of imaging, handedness, race, and cranial volume at time of imaging as covariates and adjusting for the highest parental level of education and combined parental income level as random effects. Blue areas depict regions of significant (false discovery rate corrected *p*-value < 0.05) negative association while red areas depict regions of significant (false discovery rate corrected *p*-value < 0.05) positive association.

Lastly, higher scores of the NIH Toolbox Cognitive Function Composite Score were associated with significantly lower average FA values only at the forceps minor (*β* = −2.38 × 10^−3^; *p* = 1.48 × 10^−4^) (Fig. 4a). Higer scores showed mixed associations with ND; with a negative association seen only at the forceps minor (*β* = −2.27 × 10^−3^; *p* = 3.39 × 10^−5^), while positive association was observed only at the right anterior thalamic radiations (Fig. 4a). Higher scores also correlated significantly with lower average MD and RD mainly at the corticostriate projections via the external capsule to superior frontal cortex and left superior longitudinal fasciculus, while a sole positive association was seen at the forceps minor (Fig. 4a). Finally, AD showed a negative association mainly at the corticocortical projections from inferior frontal cortex to superior frontal cortex and corticostriate projections via the external capsule to superior parietal cortex (Fig. 4a). Supplementary Tables 11–15 list the WM tracts where there were significant associations between the NIH Toolbox Cognitive Function Composite Score and diffusion metrics.

### 3.3 Association of NIH Toolbox Cognition Scores with Cortical Morphology

Higher scores from the NIH Toolbox Fluid Cognition Composite Score were associated with significantly lower cortical thickness in both hemispheres, predominantly in regions of the prefrontal cortex (e.g., medial orbitofrontal (Left: *β* = −5.25 × 10^−3^, *p* = 6.60 × 10^−3^; Right: *β* = −4.37 × 10^−3^, *p* = 1.56 × 10^−2^) and rostral middle frontal) and regions of the parietal lobe (e.g., inferior parietal, superior parietal, and precuneus) (Fig. 2b). Conversely, higher scores were also associated with significantly higher cortical surface area in both hemispheres, predominantly in regions of the prefrontal cortex (e.g., superior frontal cortex (Left: *β* = 4.55 × 10^1^, *p* = 6.72 × 10^−5^; Right: *β* = 3.59 × 10^1^, *p* = 2.79 × 10^−3^), medial orbitofrontal cortex, lateral orbitofrontal cortex) and regions of the parietal lobe (e.g., precuneus, postcentral gyrus, precentral gyrus) (Fig. 2c). Supplementary Tables 16,17 list the regions where there were significant associations between the NIH Toolbox Fluid Cognition Composite Score and cortical thickness and cortical surface area, respectively.

The Crystallized Cognition Composite Score demonstrated predominantly negative associations with cortical thickness in both hemispheres, mainly in regions of the parietal lobe (e.g., paracentral (Left: *β* = −4.48 × 10^−3^; *p* = 2.71 × 10^−2^; Right: *β* = −4.04 × 10^−3^; *p* = 3.47 × 10^−2^) (Fig. 3b). A significant positive association was also observed between the Crystallized Cognition Composite Score and cortical surface area in both hemispheres, predominantly in regions of the temporal lobe (e.g., inferior temporal cortex (Left: *β* = 1.49 × 10^1^, *p* = 2.68 × 10^−2^; Right: *β* = 2.07 × 10^1^, *p* = 2.33 × 10^−4^), fusiform cortex, banks of the superior temporal sulcus) and at the inferior parietal cortex (Fig. 3c). Supplementary Tables 18,19 list the regions where there were significant associations between the NIH Toolbox Crystallized Cognition Composite Score and cortical thickness and cortical surface area, respectively.

The NIH Toolbox Cognitive Function Composite Score was predominantly associated with lower cortical thickness, mainly in regions of the occipital lobe (e.g., cuneus (Left: *β* = −4.29 × 10^−3^, *p* = 3.69 × 10^−2^; Right: *β* = −5.01 × 10^−3^, *p* = 1.11 × 10^−2^) and the lateral occipital cortex) and regions of the parietal lobe (e.g., superior and inferior parietal cortex) (Fig. 4b). There was a strong positive association between the Cognitive Function Composite Score and cortical surface area in both hemispheres, mainly in regions of the prefrontal cortex (medial orbitofrontal (Left: *β* = 1.12 × 10^1^, *p* = 2.09 × 10^−4^; Right: *β* = 1.21 × 10^1^, *p* = 1.49 × 10^−5^), caudal middle frontal, and superior frontal cortex) and regions of the parietal lobe (fusiform cortex, inferior temporal cortex, and banks of superior temporal sulcus) (Fig. 4c). Supplementary Tables 20,21 list the regions where there were significant associations between the NIH Toolbox Cognitive Function Composite Score and cortical thickness and cortical surface area, respectively.

### 3.4 Association of NIH Toolbox Cognition Scores with Functional Connectivity

Higher values of the Fluid Cognition Composite Score were associated with mixed negative and positive associations with rs-fMRI brain connectivity. Associations were predominantly negative in relation to the auditory network and the default network. Strong positive associations were observed in intra-network connectivity within the cingulo-parietal network (*β* = 8.49 × 10^−3^; *p* = 4.40 × 10^−3^), intra-network connectivity within the dorsal attention network, and inter-network connectivity between the cingulo-parietal network and the retrosplenial temporal network (Fig. 2d). Supplementary Table 22 list all the connectivities where there were significant associations between the NIH Toolbox Fluid Cognition Composite Score and rs-fMRI.

The Crystallized Cognition Composite Score also revealed mixed negative and positive associations with rs-fMRI brain connectivity. Associations were predominantly negative in relation to the default network. Strong positive associations were observed in intra-network connectivity within the cingulo-parietal network (*β* = 7.23 × 10^−3^; *p* = 4.63 × 10^−2^), inter-network connectivity between the cingulo-parietal network and the retrosplenial temporal network, and inter-network connectivity between the fronto-parietal network and the retrosplenial temporal network (Fig. 3d). Supplementary Table 23 list all the connecitivites where there were significant associations between the NIH Toolbox Crystallized Cognition Composite Score and rs-fMRI.

Lastly, when analyzing the Cognitive Function Composite Score, we observed that higher scores were associated with mixed negative and positive associations with rs-fMRI brain connectivity. Associations were predominantly negative in relation to the auditory network. Strong positive associations were observed in intra-network connectivity within the cingulo-parietal network (*β* = 9.00 × 10^−3^; *p* = 3.31 × 10^−3^), inter-network connectivity between the cingulo-parietal network and the retrosplenial temporal network, inter-network connectivity between the fronto-parietal network and the retrosplenial temporal network, and inter-network connectivity between the dorsal attention network and fronto-parietal network (Fig. 4d). Supplementary Table 24 list all the connecitivites where there were significant associations between the NIH Toolbox Cognitive Function Composite Score and rs-fMRI.

## 4. Discussion

In this study, we examined the associations between the NIHTB-CB composite scores and various neuroimaging metrics in children. Our findings provide important insights into the neurobiological underpinnings of cognitive development in children as assessed by the NIHTB-CB. First, when analyzing the NIHTB-FCCS, we found that higher scores were associated with increased WM microstructural organization and cellular density, as indicated by our findings of increased FA and ND. This association remains after adjusting for race, ethnicity, parental income, and education. Higher NIHTB-FCCS scores also correlated with decreased average MD and RD, aligning with our findings of higher FA and ND [53]. AD also showed a negative association. Higher AD is typically a marker of WM microstructural integrity, as it measures the movement of water molecules along the direction of axonal fibers. However, during childhood neurodevelopment, the myelination of WM tracts generally restricts this movement, leading to a decrease in AD [54], as observed in our analysis (Fig. 2a).

The WM tracts that showed the strongest associations with the NIHTB-FCCS were the corticostriate projections via the external capsule to superior parietal cortex, and superior longitudinal fasciculus WM tracts. These regions are known to be involved in executive functions [55], perceptual organization, and working memory [56], which are essential components of fluid cognition. The negative associations between higher fluid cognition scores and MD and RD indicate more efficient water diffusion along WM fibers, reflecting better myelination and microstructural organization [57]. Additionally, the observed correlations with lower cortical thickness and greater cortical surface area in regions of the prefrontal cortex and parietal lobe align with the notion that cortical thinning [58–60] and surface area expansion [21] are markers of proper neurodevelopmental maturation and cognitive proficiency in children. It is important to acknowledge that in adults, reduced cortical thickness, particularly in the prefrontal cortex, has been associated with an increased risk for depression [61]. While this finding was observed in adults, it raises important considerations about the potential long-term mental health implications of cortical thinning in children as they mature. Functional connectivity MRI showed positive associations primarily at the cingulo-parietal network and the retrosplenial temporal network. These networks have been previously found to be related to executive functioning, cognitive control, attention, and memory [62–64]. In addition, impairment in these networks have been shown to cause problems with intelligence and cognitive fluctuations as those seen in dementia patients [62].

For the NIHTB-CCCS, higher scores were associated with significantly lower FA and ND values, while showing significantly positive associations for MD and RD. These opposite associations observed with DTI metrics when compared to the NIHTB-FCCS would seem to suggest that higher intelligence in children is correlated with lower overall WM microstructure. This argument seems counterintuitive since higher intelligence should be reflected with a greater not lower WM microstructure. However, we must consider the fact that crystallized intelligence, which encompasses acquired knowledge and skills, is not a great marker for children cognition as this type of intelligence develops more significantly as individuals grow older and it peaks in adulthood. This is in contrast with fluid intelligence which is the predominant form of intelligence in childhood and decreases with age [65,66]. Previous studies have demonstrated that fluid cognition is more effective when analyzing neuroimaging metrics in children compared to crystallized cognition and tend to be more sensitive to neurobiological integrity. This is evident as fluid cognitive abilities, which involve reasoning and problem-solving, show stronger associations with structural brain markers. In contrast, crystallized cognition, which encompasses accumulated knowledge and skills, is less determined by these brain metrics [9,10,67]. The NIHTB-CCCS predominantly negative associations with structural MRI cortical thickness and positive associations with cortical surface area in par with those observed NIHTB-FCCS. However, the number of significant regions associated with cortical thickness is inferior to those seen when analyzing the NIHTB-FCCS suggesting once again the superiority of fluid intelligence when studying the cognitive functioning of children. Functional connectivity MRI also showed positive associations primarily at the cingulo-parietal network and the retrosplenial temporal network, which as discussed previously have been associated with executive functioning [63] and episodic, temporary, and long-term memory [64,68,69].

Our analyses also reveal the distinctive difference between neuroimaging correlates of NIHTB-FCCS versus NIHTB-CCCS. In our study, higher NIHTB-FCCS scores (fluid cognition) were linked to increased WM microstructural integrity, as indicated by higher FA and ND. These associations were predominantly seen in WM tracts that are involved in executive functions [55], mainly the superior longitudinal fasciculus and the corticostriate projections. Additionally, increased fluid cognition correlated with cortical thinning and surface area increase in regions of the prefrontal cortex. These brain regions are important for attention, cognitive control, and working memory [70]. On the other hand, crystallized cognition, as measured by the NIHTB-CCCS scores, showed a more complex pattern of association. Higher crystallized cognition scores were associated with lower FA and ND, suggesting that more intelligent children had a more disorganized WM microstructure. These findings suggest a more complex relationship between crystallized cognition and white matter integrity during childhood. Crystallized cognition was also associated with fewer significant regions showing changes in cortical thickness and surface area compared to fluid cognition. The decrease in magnitude and extent of these correlations further supports the idea that crystallized cognition, which peaks in adulthood, may be less directly tied to childhood brain structure and neurodevelopment than fluid cognition.

Lastly, for the NIHTB-CFCS, higher scores were negatively associated with FA and AD values. Mixed positive and negative associations were seen with MD, RD, and ND. The NIHTB-CFCS, created as an effort to reflect overall cognitive performance, showed patterns consistent with both fluid and crystallized cognition. The negative associations with FA and AD, with mixed associations seen for MD, RD, and ND are consistent with a mix of the patterns observed for the NIHTB-FCCS and NIHTB-CCCS. The NIHTB-CFCS negative association with structural MRI cortical thickness and positive association with surface area mainly at the parietal lobe represent proper neurodevelopmental maturation and cognitive proficiency. Functional connectivity MRI showed positive associations in the same networks as the NIHTB-FCCS and the NIHTB-CCCS, namely the cingulo-parietal and restrosplenial temporal network. Because the NIHTB-CFCS combines both elements of fluid and crystallized cognitions, it may dilute the specific insights which would’ve provided by focusing solely on fluid cognition, which is more directly relevant to the dynamic developmental processes in children as previously alluded to.

While previous research has primarily focused on the relationship between NIHTB-CB composite scores and cognitive outcomes, our study explores the associations between these scores and neuroimaging metrics, an area that has been relatively underexplored. For example, prior studies have validated the importance of NIHTB-CB composite scores in assessing various cognitive abilities [71] but have not extensively linked these scores to specific neuroimaging metrics such as WM integrity, cortical morphology, and brain functional connectivity. Our findings on the NIHTB-FCCS are consistent with existing literature. Higher WM integrity, reflected by increased FA and ND, along with decreased MD and RD, has been associated with enhanced cognitive performance [14–16]. Additionally, other studies have linked better cognitive outcomes with thinner cortical thickness and larger surface area [21,58–60]. In contrast, the negative and mixed associations we observed for the Crystallized Cognition Composite Scores suggest a more complex relationship with neuroimaging metrics, as crystallized intelligence tends to develop more as children grow older. This finding is consistent, as discussed previously, with studies indicating that crystallized cognition might not be as strongly linked to neuroimaging metrics in children [67]. As of today, there is a lack of studies specifically analyzing the correlation of NIHTB-CB Composite Scores with neuroimaging metrics.

Our study has several notable strengths that distinguish it from prior research in the field of cognitive development and neuroimaging in children. While recently a rapidly rising number of studies have been extensively applying the NIHTB-CB composite scores to determine various cognitive outcomes [72–75], few have explored the associations between these cognitive scores and robust validated neuroimaging metrics. By focusing on this underexplored area, our research provides new insights into the structural and functional brain correlates of cognitive performance as measured by the NIHTB-CB composite scores. Previous research has demonstrated the importance of neuroimaging metrics, as measured by DTI, structural MRI, and resting-state functional MRI in providing valuable insights into cognitive functions [14–16,20–25]. Our study builds on this body of work by directly examining the relationships between NIHTB-CB composite scores and these critical neuroimaging metrics. One of the key strengths of our study is the use of the ABCD dataset, which is one of the largest and most comprehensive datasets available. This dataset includes approximately 11,800 children aged 9–10 from diverse socio-demographic backgrounds across 21 sites in the United States. The size and diversity of this sample enhance the generalizability of our findings and provide a robust foundation for examining the associations between the NIHTB-CB composite scores and neuroimaging metrics. Our study employed a multi-modal neuroimaging approach, incorporating DTI, structural MRI, and rs-fMRI. This comprehensive approach allowed us to investigate the relationships between cognitive performance and multiple aspects of brain structure and function, providing a more holistic understanding of neurocognitive development. By examining WM integrity, cortical morphology, and functional connectivity simultaneously, we were able to explore nuanced patterns that might be missed when using a single imaging modality.

While our study provides insights into the neurobiological correlations of cognitive development in children, several limitations should be acknowledged. First, from the original 11,868 sample size of the whole ABCD dataset, only 5290 subjects had all the data available and met all the quality thresholds required to perform the analysis. This substantial reduction in sample size may impact the analysis and the robustness of the findings. Second, despite the large and diverse ABCD dataset, potential biases related to site-specific recruitment strategies and demographic factors could impact the generalizability of our findings. Lastly, our focus on the NIHTB-CB composite scores may overlook specific cognitive subdomains or neurobiological interactions. Detailed analyses of individual subtests could provide more granular insights into the relationships between specific cognitive abilities and brain metrics. Despite these limitations, our study makes a crucial contribution to understanding the neurobiological mechanisms underlying cognitive development in children and explains the importance of using advanced neuroimaging techniques in cognitive development research.

## 5. Conclusions

Our study provides significant insights into the neurobiological mechanisms underlying cognitive development in children by examining the associations between NIHTB-CB composite scores and various neuroimaging metrics. Utilizing the comprehensive and diverse ABCD dataset, we found that higher NIH Toolbox Fluid Cognition Composite Scores were strongly associated with expected neuroimaging markers of cognitive development, such as better WM integrity, lower cortical thickness, and greater cortical surface area. These findings underscore the relevance of the NIH Toolbox Fluid Cognition Composite Score as a key marker for cognitive development in children. In contrast, the NIH Toolbox Crystallized Cognition Composite Scores showed more complex and less consistent associations with neuroimaging metrics, which may be caused by the fact that crystallized intelligence develops more significantly with age and may not be as useful when assessing children cognitive development. The NIH Toolbox Cognitive Function Composite Scores, while useful, tend to dilute the specific insights provided by focusing solely on fluid or crystallized cognition. The combination of these domains in the total score can obscure the distinct neurobiological mechanisms underpinning each cognitive ability. Given the robust associations observed with neuroimaging metrics, when utilizing the NIH Toolbox Fluid Cognition Composite Score, it appears that this composite score provides a comprehensive reflection of neurodevelopmental and neurocognitive processes and should be considered a valuable tool in assessments of children’s cognitive abilities. This focus can enhance the precision of neurodevelopmental assessments and inform targeted interventions and educational strategies to support cognitive growth during children’s critical developmental periods.

## Supplementary Material

Supplementary

## Figures and Tables

**Table 1.** Demographic characteristics of subjects included in the study.

Characteristic	(N = 5290)
Age, mean (SD), years	9.9 (0.6)
BMI *z* score, mean (SD)	0.33 (1.14)
Sex, number (%)	
Male	2574 (48.66)
Female	2716 (51.34)
Race and Ethnicity, number (%)	
White	3122 (59.02)
Black	590 (11.15)
Asian	105 (1.98)
Hispanic	1060 (20.04)
Multiracial or Other	413 (7.81)
Parental Education, number (%)	
<High school	191 (3.61)
High school or GED	380 (7.18)
Some college	1244 (23.52)
Bachelor’s degree	1435 (27.13)
Postgraduate	2040 (38.56)
Handedness, number (%)	
Right	4328 (81.81)
Left	357 (6.75)
Mixed	605 (11.44)
Family income, number (%)	
<$5000	156 (2.95)
$5000–$11,999	158 (2.99)
$12,000–$15,999	121 (2.29)
$16,000–$24,999	215 (4.06)
$25,000–$34,999	303 (5.73)
$35,000–$49,999	405 (7.66)
$50,000–$74,999	711 (13.44)
$75,000–$99,999	809 (15.29)
$100,000–$199,999	1745 (32.99)
≥$200,000	667 (12.61)

Note: Data are reported as mean (standard deviation), or number of subjects (percentage). BMI, Body Mass Index; GED, General Education Development.

## Data Availability

Data used in the preparation of this article were obtained from the Adolescent Brain Cognitive Development (ABCD) Study (https://abcdstudy.org), held in the NIMH Data Archive (NDA). This is a multisite, longitudinal study designed to recruit more than 10,000 children aged 9–10 and follow them over 10 years into early adulthood.
