# Supplementary material for: Neuroimaging Correlates of the NIH-Toolbox-Driven Cognitive Metrics in Children
Source: J Integr Neurosci. Author manuscript; Available in PMC 2025 Feb 25. (PMC11851640; doi:10.31083/j.jin2312217)
Supplement: Supplementary [file NIHMS2054769-supplement-Supplementary.docx]

**Supplementary Table 1**. Mixed linear model analysis of the association of the NIH Toolbox Fluid Cognition Composite Score with fractional anisotropy (FA) values at baseline after controlling for subjects’ age at time of imaging, sex, BMI z-score at time of imaging, handedness, race, and cranial volume at time of imaging as covariates and adjusting for the highest parental level of education and combined parental income level as random effects.

| **White matter tract** | **Coefficient (95% confidence interval)** | **Original p-value** | **Corrected p-value** |
| --- | --- | --- | --- |
| Right Anterior Thalamic Radiations | 1.16E-03 (2.86E-04 to 2.04E-03) | 9.41E-03 | 2.33E-02 |
| Right Superior Longitudinal Fasiculus | 9.58E-04 (1.24E-04 to 1.79E-03) | 2.44E-02 | 5.59E-02 |
| Left Superior Longitudinal Fasiculus | 1.12E-03 (2.69E-04 to 1.97E-03) | 9.84E-03 | 2.43E-02 |
| Right Temporal Superior Longitudinal Fasiculus | 9.60E-04 (1.20E-04 to 1.80E-03) | 2.50E-02 | 5.73E-02 |
| Left Temporal Superior Longitudinal Fasiculus | 1.13E-03 (2.61E-04 to 1.99E-03) | 1.08E-02 | 2.63E-02 |
| Right Parietal Superior Longitudinal Fasiculus | 9.06E-04 (5.74E-05 to 1.75E-03) | 3.64E-02 | 8.06E-02 |
| Left Parietal Superior Longitudinal Fasiculus | 1.01E-03 (1.54E-04 to 1.87E-03) | 2.08E-02 | 4.85E-02 |
| Right Superior Corticostriate | 1.03E-03 (1.37E-04 to 1.93E-03) | 2.39E-02 | 5.52E-02 |
| Left Superior Corticostriate | 9.92E-04 (2.52E-04 to 1.73E-03) | 8.65E-03 | 2.16E-02 |
| Left Corticostriate projections via the external capsule to superior frontal cortex | 9.56E-04 (2.05E-04 to 1.71E-03) | 1.26E-02 | 3.04E-02 |
| Right Corticostriate projections via the external capsule to superior parietal cortex | 1.07E-03 (1.40E-04 to 2.00E-03) | 2.41E-02 | 5.55E-02 |
| Left Corticostriate projections via the external capsule to superior parietal cortex | 1.16E-03 (3.32E-04 to 2.00E-03) | 6.11E-03 | 1.54E-02 |

Both the original and the false-discovery-rate-corrected p-values for paired t-tests are provided. Only analyses with significant uncorrected p-values are included. values are shown.

**Supplementary Table 2**. Mixed linear model analysis of the association of the NIH Toolbox Fluid Cognition Composite Score with mean diffusivity (MD) values at baseline after controlling for subjects’ age at time of imaging, sex, BMI z-score at time of imaging, handedness, race, and cranial volume at time of imaging as covariates and adjusting for the highest parental level of education and combined parental income level as random effects.

| **White matter tract** | **Coefficient (95% confidence interval)** | **Original p-value** | **Corrected p-value** |
| --- | --- | --- | --- |
| Right Anterior Thalamic Radiations | -6.04E-04 (-1.15E-03 to -5.67E-05) | 3.05E-02 | 6.89E-02 |
| Left Inferior Longitudinal Fasiculus | -6.41E-04 (-1.21E-03 to -7.58E-05) | 2.62E-02 | 5.96E-02 |
| Right Superior Longitudinal Fasiculus | -7.01E-04 (-1.15E-03 to -2.53E-04) | 2.15E-03 | 5.70E-03 |
| Left Superior Longitudinal Fasiculus | -8.71E-04 (-1.33E-03 to -4.11E-04) | 2.10E-04 | 6.25E-04 |
| Right Temporal Superior Longitudinal Fasiculus | -7.36E-04 (-1.19E-03 to -2.82E-04) | 1.47E-03 | 4.00E-03 |
| Left Temporal Superior Longitudinal Fasiculus | -7.79E-04 (-1.25E-03 to -3.13E-04) | 1.05E-03 | 2.90E-03 |
| Right Parietal Superior Longitudinal Fasiculus | -7.20E-04 (-1.17E-03 to -2.72E-04) | 1.64E-03 | 4.44E-03 |
| Left Parietal Superior Longitudinal Fasiculus | -1.02E-03 (-1.48E-03 to -5.64E-04) | 1.22E-05 | 4.05E-05 |
| Right Superior Corticostriate | -8.43E-04 (-1.24E-03 to -4.42E-04) | 3.70E-05 | 1.17E-04 |
| Left Superior Corticostriate | -9.12E-04 (-1.32E-03 to -5.07E-04) | 1.01E-05 | 3.36E-05 |
| Right Corticostriate projections via the external capsule to superior frontal cortex | -8.63E-04 (-1.25E-03 to -4.75E-04) | 1.34E-05 | 4.43E-05 |
| Left Corticostriate projections via the external capsule to superior frontal cortex | -8.67E-04 (-1.27E-03 to -4.66E-04) | 2.22E-05 | 7.16E-05 |
| Right Corticostriate projections via the external capsule to superior parietal cortex | -8.87E-04 (-1.31E-03 to -4.67E-04) | 3.49E-05 | 1.11E-04 |
| Left Corticostriate projections via the external capsule to superior parietal cortex | -1.05E-03 (-1.48E-03 to -6.26E-04) | 1.45E-06 | 5.22E-06 |
| Right Corticocortical projections from inferior frontal cortex to superior frontal cortex | -9.55E-04 (-1.39E-03 to -5.19E-04) | 1.72E-05 | 5.59E-05 |
| Left Corticocortical projections from inferior frontal cortex to superior frontal cortex | -9.88E-04 (-1.44E-03 to -5.40E-04) | 1.56E-05 | 5.11E-05 |

Both the original and the false-discovery-rate-corrected p-values for paired t-tests are provided. Only analyses with significant uncorrected p-values are included. values are shown.

**Supplementary Table 3**. Mixed linear model analysis of the association of the NIH Toolbox Fluid Cognition Composite Score with radial diffusivity (RD) values at baseline after controlling for subjects’ age at time of imaging, sex, BMI z-score at time of imaging, handedness, race, and cranial volume at time of imaging as covariates and adjusting for the highest parental level of education and combined parental income level as random effects.

| **White matter tract** | **Coefficient (95% confidence interval)** | **Original p-value** | **Corrected p-value** |
| --- | --- | --- | --- |
| Right Cingulate Cingulum | -8.35E-04 (-1.63E-03 to -4.19E-05) | 3.90E-02 | 8.60E-02 |
| Right Corticospinal/pyramidal | -4.35E-04 (-8.68E-04 to -2.35E-06) | 4.88E-02 | 1.06E-01 |
| Left Corticospinal/pyramidal | -5.91E-04 (-1.03E-03 to -1.49E-04) | 8.82E-03 | 2.19E-02 |
| Right Anterior Thalamic Radiations | -9.19E-04 (-1.41E-03 to -4.29E-04) | 2.38E-04 | 7.02E-04 |
| Left Anterior Thalamic Radiations | -6.67E-04 (-1.17E-03 to -1.60E-04) | 9.92E-03 | 2.45E-02 |
| Right Inferior Longitudinal Fasiculus | -6.26E-04 (-1.21E-03 to -4.56E-05) | 3.45E-02 | 7.69E-02 |
| Left Inferior Longitudinal Fasiculus | -8.34E-04 (-1.43E-03 to -2.40E-04) | 5.88E-03 | 1.48E-02 |
| Forceps Major | -9.05E-04 (-1.67E-03 to -1.45E-04) | 1.96E-02 | 4.59E-02 |
| Right Superior Longitudinal Fasiculus | -8.86E-04 (-1.40E-03 to -3.73E-04) | 7.08E-04 | 2.00E-03 |
| Left Superior Longitudinal Fasiculus | -1.04E-03 (-1.57E-03 to -5.07E-04) | 1.26E-04 | 3.84E-04 |
| Right Temporal Superior Longitudinal Fasiculus | -9.05E-04 (-1.42E-03 to -3.86E-04) | 6.26E-04 | 1.77E-03 |
| Left Temporal Superior Longitudinal Fasiculus | -9.94E-04 (-1.53E-03 to -4.57E-04) | 2.88E-04 | 8.45E-04 |
| Right Parietal Superior Longitudinal Fasiculus | -8.78E-04 (-1.40E-03 to -3.59E-04) | 9.09E-04 | 2.52E-03 |
| Left Parietal Superior Longitudinal Fasiculus | -1.07E-03 (-1.61E-03 to -5.37E-04) | 8.73E-05 | 2.69E-04 |
| Right Superior Corticostriate | -9.55E-04 (-1.39E-03 to -5.21E-04) | 1.66E-05 | 5.41E-05 |
| Left Superior Corticostriate | -9.93E-04 (-1.40E-03 to -5.89E-04) | 1.41E-06 | 5.09E-06 |
| Right Corticostriate projections via the external capsule to superior frontal cortex | -9.12E-04 (-1.33E-03 to -4.96E-04) | 1.75E-05 | 5.68E-05 |
| Left Corticostriate projections via the external capsule to superior frontal cortex | -9.45E-04 (-1.36E-03 to -5.33E-04) | 7.00E-06 | 2.36E-05 |
| Right Corticostriate projections via the external capsule to superior parietal cortex | -9.96E-04 (-1.45E-03 to -5.37E-04) | 2.06E-05 | 6.65E-05 |
| Left Corticostriate projections via the external capsule to superior parietal cortex | -1.14E-03 (-1.58E-03 to -6.99E-04) | 4.33E-07 | 1.60E-06 |
| Right Corticocortical projections from inferior frontal cortex to superior frontal cortex | -9.26E-04 (-1.41E-03 to -4.41E-04) | 1.82E-04 | 5.47E-04 |
| Left Corticocortical projections from inferior frontal cortex to superior frontal cortex | -9.45E-04 (-1.45E-03 to -4.42E-04) | 2.30E-04 | 6.82E-04 |

Both the original and the false-discovery-rate-corrected p-values for paired t-tests are provided. Only analyses with significant uncorrected p-values are included. values are shown.

**Supplementary Table 4**. Mixed linear model analysis of the association of the NIH Toolbox Fluid Cognition Composite Score with axial diffusivity (AD) values at baseline after controlling for subjects’ age at time of imaging, sex, BMI z-score at time of imaging, handedness, race, and cranial volume at time of imaging as covariates and adjusting for the highest parental level of education and combined parental income level as random effects.

| **White matter tract** | **Coefficient (95% confidence interval)** | **Original p-value** | **Corrected p-value** |
| --- | --- | --- | --- |
| Left Parietal Superior Longitudinal Fasiculus | -8.88E-04 (-1.60E-03 to -1.73E-04) | 1.49E-02 | 3.55E-02 |
| Right Corticocortical projections from inferior frontal cortex to superior frontal cortex | -1.01E-03 (-1.71E-03 to -3.05E-04) | 4.93E-03 | 1.26E-02 |
| Left Corticocortical projections from inferior frontal cortex to superior frontal cortex | -1.06E-03 (-1.76E-03 to -3.51E-04) | 3.32E-03 | 8.60E-03 |

Both the original and the false-discovery-rate-corrected p-values for paired t-tests are provided. Only analyses with significant uncorrected p-values are included. values are shown.

**Supplementary Table 5**. Mixed linear model analysis of the association of the NIH Toolbox Fluid Cognition Composite Score with neurite density (ND) values at baseline after controlling for subjects’ age at time of imaging, sex, BMI z-score at time of imaging, handedness, race, and cranial volume at time of imaging as covariates and adjusting for the highest parental level of education and combined parental income level as random effects.

| **White matter tract** | **Coefficient (95% confidence interval)** | **Original p-value** | **Corrected p-value** |
| --- | --- | --- | --- |
| Left Fornix | 1.16E-03 (2.94E-04 to 2.02E-03) | 8.62E-03 | 2.02E-02 |
| Left Corticospinal/pyramidal | 5.40E-04 (1.84E-05 to 1.06E-03) | 4.24E-02 | 8.98E-02 |
| Right Anterior Thalamic Radiations | 1.58E-03 (8.19E-04 to 2.35E-03) | 4.84E-05 | 1.48E-04 |
| Left Anterior Thalamic Radiations | 1.08E-03 (3.00E-04 to 1.85E-03) | 6.60E-03 | 1.58E-02 |
| Left Inferior Longitudinal Fasiculus | 8.79E-04 (8.70E-05 to 1.67E-03) | 2.96E-02 | 6.39E-02 |
| Forceps Major | 1.04E-03 (1.42E-04 to 1.94E-03) | 2.32E-02 | 5.08E-02 |
| Right Superior Longitudinal Fasiculus | 9.01E-04 (2.18E-04 to 1.59E-03) | 9.78E-03 | 2.27E-02 |
| Left Superior Longitudinal Fasiculus | 1.11E-03 (4.15E-04 to 1.80E-03) | 1.72E-03 | 4.46E-03 |
| Right Temporal Superior Longitudinal Fasiculus | 1.00E-03 (3.26E-04 to 1.68E-03) | 3.66E-03 | 9.01E-03 |
| Left Temporal Superior Longitudinal Fasiculus | 1.04E-03 (3.52E-04 to 1.73E-03) | 3.05E-03 | 7.54E-03 |
| Right Parietal Superior Longitudinal Fasiculus | 8.52E-04 (1.44E-04 to 1.56E-03) | 1.84E-02 | 4.08E-02 |
| Left Parietal Superior Longitudinal Fasiculus | 1.16E-03 (4.16E-04 to 1.91E-03) | 2.26E-03 | 5.78E-03 |
| Right Superior Corticostriate | 1.22E-03 (5.30E-04 to 1.91E-03) | 5.21E-04 | 1.49E-03 |
| Left Superior Corticostriate | 1.15E-03 (5.65E-04 to 1.73E-03) | 1.14E-04 | 3.43E-04 |
| Right Corticostriate projections via the external capsule to superior frontal cortex | 8.88E-04 (2.22E-04 to 1.55E-03) | 8.94E-03 | 2.09E-02 |
| Left Corticostriate projections via the external capsule to superior frontal cortex | 1.03E-03 (4.18E-04 to 1.65E-03) | 9.84E-04 | 2.73E-03 |
| Right Corticostriate projections via the external capsule to superior parietal cortex | 1.27E-03 (5.64E-04 to 1.97E-03) | 4.13E-04 | 1.20E-03 |
| Left Corticostriate projections via the external capsule to superior parietal cortex | 1.36E-03 (7.22E-04 to 2.00E-03) | 2.95E-05 | 9.21E-05 |
| Right Corticocortical projections from inferior frontal cortex to superior frontal cortex | 8.08E-04 (1.08E-04 to 1.51E-03) | 2.36E-02 | 5.13E-02 |
| Left Corticocortical projections from inferior frontal cortex to superior frontal cortex | 8.89E-04 (1.72E-04 to 1.61E-03) | 1.51E-02 | 3.45E-02 |

Both the original and the false-discovery-rate-corrected p-values for paired t-tests are provided. Only analyses with significant uncorrected p-values are included. values are shown.

**Supplementary Table 6**. Mixed linear model analysis of the association of the NIH Toolbox Crystallized Cognition Composite Score with fractional anisotropy (FA) values at baseline after controlling for subjects’ age at time of imaging, sex, BMI z-score at time of imaging, handedness, race, and cranial volume at time of imaging as covariates and adjusting for the highest parental level of education and combined parental income level as random effects.

| **White matter tract** | **Coefficient (95% confidence interval)** | **Original p-value** | **Corrected p-value** |
| --- | --- | --- | --- |
| Left Cingulate Cingulum | -2.34E-03 (-3.73E-03 to -9.46E-04) | 9.86E-04 | 2.80E-03 |
| Right Parahippocampal Cingulum | -1.68E-03 (-2.87E-03 to -4.81E-04) | 5.98E-03 | 1.55E-02 |
| Left Corticospinal/pyramidal | -9.91E-04 (-1.82E-03 to -1.62E-04) | 1.91E-02 | 4.62E-02 |
| Right Uncinate | -1.40E-03 (-2.37E-03 to -4.36E-04) | 4.47E-03 | 1.17E-02 |
| Left Uncinate | -1.04E-03 (-2.09E-03 to -3.66E-06) | 4.92E-02 | 1.09E-01 |
| Right Inferior Longitudinal Fasiculus | -1.03E-03 (-1.94E-03 to -1.26E-04) | 2.56E-02 | 6.03E-02 |
| Forceps Major | -2.11E-03 (-3.22E-03 to -9.96E-04) | 2.00E-04 | 6.09E-04 |
| Forceps Minor | -3.00E-03 (-4.13E-03 to -1.86E-03) | 2.28E-07 | 8.50E-07 |
| Corpus Callosum | -1.96E-03 (-2.85E-03 to -1.06E-03) | 1.79E-05 | 5.85E-05 |
| Right Corticostriate projections to inferior frontal cortex | -1.19E-03 (-2.07E-03 to -3.19E-04) | 7.50E-03 | 1.92E-02 |
| Left Corticostriate projections to inferior frontal cortex | -1.15E-03 (-2.00E-03 to -2.98E-04) | 8.14E-03 | 2.07E-02 |
| Right Corticocortical projections from inferior frontal cortex to superior frontal cortex | -9.03E-04 (-1.69E-03 to -1.15E-04) | 2.46E-02 | 5.83E-02 |
| Left Corticocortical projections from inferior frontal cortex to superior frontal cortex | -1.02E-03 (-1.81E-03 to -2.36E-04) | 1.08E-02 | 2.67E-02 |

Both the original and the false-discovery-rate-corrected p-values for paired t-tests are provided. Only analyses with significant uncorrected p-values are included. values are shown.

**Supplementary Table 7**. Mixed linear model analysis of the association of the NIH Toolbox Crystallized Cognition Composite Score with mean diffusivity (MD) values at baseline after controlling for subjects’ age at time of imaging, sex, BMI z-score at time of imaging, handedness, race, and cranial volume at time of imaging as covariates and adjusting for the highest parental level of education and combined parental income level as random effects.

| **White matter tract** | **Coefficient (95% confidence interval)** | **Original p-value** | **Corrected p-value** |
| --- | --- | --- | --- |
| Left Uncinate | 7.14E-04 (1.03E-04 to 1.32E-03) | 2.20E-02 | 5.22E-02 |
| Forceps Major | 6.49E-04 (4.85E-06 to 1.29E-03) | 4.83E-02 | 1.07E-01 |
| Forceps Minor | 1.46E-03 (7.35E-04 to 2.18E-03) | 7.52E-05 | 2.36E-04 |

Both the original and the false-discovery-rate-corrected p-values for paired t-tests are provided. Only analyses with significant uncorrected p-values are included. values are shown.

\

**Supplementary Table 8**. Mixed linear model analysis of the association of the NIH Toolbox Crystallized Cognition Composite Score with radial diffusivity (RD) values at baseline after controlling for subjects’ age at time of imaging, sex, BMI z-score at time of imaging, handedness, race, and cranial volume at time of imaging as covariates and adjusting for the highest parental level of education and combined parental income level as random effects.

| **White matter tract** | **Coefficient (95% confidence interval)** | **Original p-value** | **Corrected p-value** |
| --- | --- | --- | --- |
| Left Cingulate Cingulum | 1.09E-03 (3.08E-04 to 1.88E-03) | 6.35E-03 | 1.64E-02 |
| Right Parahippocampal Cingulum | 9.07E-04 (9.39E-05 to 1.72E-03) | 2.88E-02 | 6.68E-02 |
| Left Parahippocampal Cingulum | 9.96E-04 (1.97E-04 to 1.80E-03) | 1.45E-02 | 3.57E-02 |
| Left Corticospinal/pyramidal | 4.81E-04 (3.10E-05 to 9.31E-04) | 3.62E-02 | 8.25E-02 |
| Right Uncinate | 1.06E-03 (3.56E-04 to 1.76E-03) | 3.14E-03 | 8.44E-03 |
| Left Uncinate | 9.81E-04 (2.65E-04 to 1.70E-03) | 7.27E-03 | 1.87E-02 |
| Forceps Major | 1.43E-03 (6.59E-04 to 2.20E-03) | 2.80E-04 | 8.39E-04 |
| Forceps Minor | 2.27E-03 (1.45E-03 to 3.08E-03) | 5.28E-08 | 2.05E-07 |
| Corpus Callosum | 1.16E-03 (5.70E-04 to 1.75E-03) | 1.16E-04 | 3.57E-04 |
| Right Corticostriate projections to inferior frontal cortex | 8.67E-04 (2.23E-04 to 1.51E-03) | 8.28E-03 | 2.10E-02 |
| Left Corticostriate projections to inferior frontal cortex | 8.22E-04 (2.19E-04 to 1.42E-03) | 7.53E-03 | 1.92E-02 |

Both the original and the false-discovery-rate-corrected p-values for paired t-tests are provided. Only analyses with significant uncorrected p-values are included. values are shown.

**Supplementary Table 9**. Mixed linear model analysis of the association of the NIH Toolbox Crystallized Cognition Composite Score with axial diffusivity (AD) values at baseline after controlling for subjects’ age at time of imaging, sex, BMI z-score at time of imaging, handedness, race, and cranial volume at time of imaging as covariates and adjusting for the highest parental level of education and combined parental income level as random effects.

| **White matter tract** | **Coefficient (95% confidence interval)** | **Original p-value** | **Corrected p-value** |
| --- | --- | --- | --- |
| Left Cingulate Cingulum | -1.66E-03 (-3.15E-03 to -1.81E-04) | 2.79E-02 | 6.52E-02 |
| Right Superior Longitudinal Fasiculus | -8.23E-04 (-1.57E-03 to -7.32E-05) | 3.15E-02 | 7.28E-02 |
| Right Temporal Superior Longitudinal Fasiculus | -8.12E-04 (-1.59E-03 to -3.44E-05) | 4.07E-02 | 9.22E-02 |
| Right Parietal Superior Longitudinal Fasiculus | -9.10E-04 (-1.66E-03 to -1.61E-04) | 1.73E-02 | 4.21E-02 |
| Right Superior Corticostriate | -1.02E-03 (-1.99E-03 to -6.20E-05) | 3.70E-02 | 8.41E-02 |
| Right Corticostriate projections via the external capsule to superior parietal cortex | -1.27E-03 (-2.28E-03 to -2.68E-04) | 1.31E-02 | 3.23E-02 |
| Left Corticostriate projections via the external capsule to superior parietal cortex | -1.05E-03 (-1.98E-03 to -1.28E-04) | 2.58E-02 | 6.07E-02 |
| Right Corticocortical projections from inferior frontal cortex to superior frontal cortex | -9.79E-04 (-1.69E-03 to -2.66E-04) | 7.08E-03 | 1.82E-02 |
| Left Corticocortical projections from inferior frontal cortex to superior frontal cortex | -1.00E-03 (-1.72E-03 to -2.87E-04) | 6.03E-03 | 1.56E-02 |

Both the original and the false-discovery-rate-corrected p-values for paired t-tests are provided. Only analyses with significant uncorrected p-values are included. values are shown.

**Supplementary Table 10**. Mixed linear model analysis of the association of the NIH Toolbox Crystallized Cognition Composite Score with neurite density (ND) values at baseline after controlling for subjects’ age at time of imaging, sex, BMI z-score at time of imaging, handedness, race, and cranial volume at time of imaging as covariates and adjusting for the highest parental level of education and combined parental income level as random effects.

| **White matter tract** | **Coefficient (95% confidence interval)** | **Original p-value** | **Corrected p-value** |
| --- | --- | --- | --- |
| Left Cingulate Cingulum | -2.05E-03 (-3.30E-03 to -7.96E-04) | 1.35E-03 | 3.65E-03 |
| Right Parahippocampal Cingulum | -1.51E-03 (-2.70E-03 to -3.25E-04) | 1.26E-02 | 2.93E-02 |
| Right Corticospinal/pyramidal | -6.20E-04 (-1.15E-03 to -9.46E-05) | 2.07E-02 | 4.74E-02 |
| Left Corticospinal/pyramidal | -7.05E-04 (-1.23E-03 to -1.75E-04) | 9.09E-03 | 2.21E-02 |
| Right Uncinate | -1.51E-03 (-2.51E-03 to -5.04E-04) | 3.22E-03 | 8.19E-03 |
| Left Uncinate | -1.15E-03 (-2.18E-03 to -1.14E-04) | 2.95E-02 | 6.54E-02 |
| Right Inferior Longitudinal Fasiculus | -9.56E-04 (-1.77E-03 to -1.40E-04) | 2.17E-02 | 4.91E-02 |
| Right Inferior-fronto-occipital Fasiculus | -8.16E-04 (-1.63E-03 to -1.51E-06) | 4.96E-02 | 1.07E-01 |
| Forceps Major | -1.66E-03 (-2.57E-03 to -7.47E-04) | 3.66E-04 | 1.07E-03 |
| Forceps Minor | -2.80E-03 (-3.80E-03 to -1.80E-03) | 3.97E-08 | 1.47E-07 |
| Corpus Callosum | -1.49E-03 (-2.19E-03 to -7.93E-04) | 2.88E-05 | 8.86E-05 |
| Right Corticostriate projections to inferior frontal cortex | -1.23E-03 (-2.19E-03 to -2.65E-04) | 1.25E-02 | 2.92E-02 |
| Left Corticostriate projections to inferior frontal cortex | -1.23E-03 (-2.13E-03 to -3.23E-04) | 7.87E-03 | 1.93E-02 |
| Right Corticocortical projections from inferior frontal cortex to superior frontal cortex | -8.02E-04 (-1.51E-03 to -9.15E-05) | 2.69E-02 | 6.01E-02 |
| Left Corticocortical projections from inferior frontal cortex to superior frontal cortex | -8.63E-04 (-1.59E-03 to -1.35E-04) | 2.02E-02 | 4.63E-02 |

Both the original and the false-discovery-rate-corrected p-values for paired t-tests are provided. Only analyses with significant uncorrected p-values are included. values are shown.

**Supplementary Table 11**. Mixed linear model analysis of the association of the NIH Toolbox Total Cognitive Function Composite Score with fractional anisotropy (FA) values at baseline after controlling for subjects’ age at time of imaging, sex, BMI z-score at time of imaging, handedness, race, and cranial volume at time of imaging as covariates and adjusting for the highest parental level of education and combined parental income level as random effects.

| **White matter tract** | **Coefficient (95% confidence interval)** | **Original p-value** | **Corrected p-value** |
| --- | --- | --- | --- |
| Right Uncinate | -9.80E-04 (-1.95E-03 to -1.01E-05) | 4.77E-02 | 1.05E-01 |
| Forceps Minor | -2.38E-03 (-3.52E-03 to -1.24E-03) | 4.28E-05 | 1.48E-04 |
| Corpus Callosum | -9.46E-04 (-1.84E-03 to -4.71E-05) | 3.91E-02 | 8.86E-02 |
| Right Corticostriate projections to inferior frontal cortex | -9.72E-04 (-1.85E-03 to -9.37E-05) | 3.01E-02 | 7.00E-02 |
| Left Corticostriate projections to inferior frontal cortex | -8.68E-04 (-1.72E-03 to -1.35E-05) | 4.65E-02 | 1.03E-01 |

Both the original and the false-discovery-rate-corrected p-values for paired t-tests are provided. Only analyses with significant uncorrected p-values are included. values are shown.

**Supplementary Table 12**. Mixed linear model analysis of the association of the NIH Toolbox Total Cognitive Function Composite Score with mean diffusivity (MD) values at baseline after controlling for subjects’ age at time of imaging, sex, BMI z-score at time of imaging, handedness, race, and cranial volume at time of imaging as covariates and adjusting for the highest parental level of education and combined parental income level as random effects.

| **White matter tract** | **Coefficient (95% confidence interval)** | **Original p-value** | **Corrected p-value** |
| --- | --- | --- | --- |
| Forceps Minor | 1.08E-03 (3.61E-04 to 1.80E-03) | 3.27E-03 | 9.26E-03 |
| Right Superior Longitudinal Fasiculus | -5.79E-04 (-1.04E-03 to -1.23E-04) | 1.28E-02 | 3.24E-02 |
| Left Superior Longitudinal Fasiculus | -6.72E-04 (-1.14E-03 to -2.03E-04) | 5.00E-03 | 1.37E-02 |
| Right Temporal Superior Longitudinal Fasiculus | -5.38E-04 (-1.00E-03 to -7.69E-05) | 2.22E-02 | 5.34E-02 |
| Left Temporal Superior Longitudinal Fasiculus | -5.85E-04 (-1.06E-03 to -1.09E-04) | 1.60E-02 | 3.98E-02 |
| Right Parietal Superior Longitudinal Fasiculus | -6.16E-04 (-1.07E-03 to -1.59E-04) | 8.24E-03 | 2.19E-02 |
| Left Parietal Superior Longitudinal Fasiculus | -8.43E-04 (-1.31E-03 to -3.76E-04) | 4.00E-04 | 1.28E-03 |
| Right Superior Corticostriate | -6.84E-04 (-1.09E-03 to -2.75E-04) | 1.03E-03 | 3.14E-03 |
| Left Superior Corticostriate | -6.73E-04 (-1.09E-03 to -2.60E-04) | 1.40E-03 | 4.19E-03 |
| Right Corticostriate projections via the external capsule to superior frontal cortex | -7.34E-04 (-1.13E-03 to -3.38E-04) | 2.79E-04 | 9.11E-04 |
| Left Corticostriate projections via the external capsule to superior frontal cortex | -6.55E-04 (-1.06E-03 to -2.47E-04) | 1.65E-03 | 4.88E-03 |
| Right Corticostriate projections via the external capsule to superior parietal cortex | -7.15E-04 (-1.14E-03 to -2.88E-04) | 1.05E-03 | 3.18E-03 |
| Left Corticostriate projections via the external capsule to superior parietal cortex | -8.08E-04 (-1.25E-03 to -3.71E-04) | 2.95E-04 | 9.60E-04 |
| Right Corticocortical projections from inferior frontal cortex to superior frontal cortex | -6.87E-04 (-1.13E-03 to -2.44E-04) | 2.40E-03 | 6.93E-03 |
| Left Corticocortical projections from inferior frontal cortex to superior frontal cortex | -7.01E-04 (-1.16E-03 to -2.44E-04) | 2.64E-03 | 7.58E-03 |

Both the original and the false-discovery-rate-corrected p-values for paired t-tests are provided. Only analyses with significant uncorrected p-values are included. values are shown.

**Supplementary Table 13**. Mixed linear model analysis of the association of the NIH Toolbox Total Cognitive Function Composite Score with radial diffusivity (RD) values at baseline after controlling for subjects’ age at time of imaging, sex, BMI z-score at time of imaging, handedness, race, and cranial volume at time of imaging as covariates and adjusting for the highest parental level of education and combined parental income level as random effects.

| **White matter tract** | **Coefficient (95% confidence interval)** | **Original p-value** | **Corrected p-value** |
| --- | --- | --- | --- |
| Right Anterior Thalamic Radiations | -6.47E-04 (-1.15E-03 to -1.47E-04) | 1.12E-02 | 2.87E-02 |
| Forceps Minor | 1.74E-03 (9.23E-04 to 2.56E-03) | 3.04E-05 | 1.07E-04 |
| Left Superior Longitudinal Fasiculus | -6.44E-04 (-1.18E-03 to -1.04E-04) | 1.95E-02 | 4.76E-02 |
| Left Temporal Superior Longitudinal Fasiculus | -5.50E-04 (-1.10E-03 to -2.26E-06) | 4.91E-02 | 1.08E-01 |
| Left Parietal Superior Longitudinal Fasiculus | -7.90E-04 (-1.34E-03 to -2.44E-04) | 4.58E-03 | 1.26E-02 |
| Right Superior Corticostriate | -5.00E-04 (-9.43E-04 to -5.65E-05) | 2.71E-02 | 6.39E-02 |
| Left Superior Corticostriate | -4.92E-04 (-9.04E-04 to -8.08E-05) | 1.90E-02 | 4.65E-02 |
| Right Corticostriate projections via the external capsule to superior frontal cortex | -5.94E-04 (-1.02E-03 to -1.70E-04) | 6.02E-03 | 1.63E-02 |
| Left Corticostriate projections via the external capsule to superior frontal cortex | -5.05E-04 (-9.26E-04 to -8.48E-05) | 1.85E-02 | 4.53E-02 |
| Left Corticostriate projections via the external capsule to superior parietal cortex | -5.86E-04 (-1.04E-03 to -1.34E-04) | 1.10E-02 | 2.84E-02 |

Both the original and the false-discovery-rate-corrected p-values for paired t-tests are provided. Only analyses with significant uncorrected p-values are included. values are shown.

**Supplementary Table 14**. Mixed linear model analysis of the association of the NIH Toolbox Total Cognitive Function Composite Score with axial diffusivity (AD) values at baseline after controlling for subjects’ age at time of imaging, sex, BMI z-score at time of imaging, handedness, race, and cranial volume at time of imaging as covariates and adjusting for the highest parental level of education and combined parental income level as random effects.

| **White matter tract** | **Coefficient (95% confidence interval)** | **Original p-value** | **Corrected p-value** |
| --- | --- | --- | --- |
| Right Superior Longitudinal Fasiculus | -7.88E-04 (-1.54E-03 to -3.44E-05) | 4.04E-02 | 9.13E-02 |
| Right Temporal Superior Longitudinal Fasiculus | -7.84E-04 (-1.57E-03 to -2.54E-06) | 4.93E-02 | 1.08E-01 |
| Right Parietal Superior Longitudinal Fasiculus | -8.86E-04 (-1.64E-03 to -1.34E-04) | 2.10E-02 | 5.08E-02 |
| Left Parietal Superior Longitudinal Fasiculus | -9.44E-04 (-1.67E-03 to -2.16E-04) | 1.11E-02 | 2.86E-02 |
| Right Superior Corticostriate | -1.05E-03 (-2.01E-03 to -7.97E-05) | 3.39E-02 | 7.80E-02 |
| Left Superior Corticostriate | -1.03E-03 (-1.87E-03 to -1.91E-04) | 1.61E-02 | 4.01E-02 |
| Right Corticostriate projections via the external capsule to superior frontal cortex | -1.01E-03 (-1.90E-03 to -1.19E-04) | 2.63E-02 | 6.23E-02 |
| Left Corticostriate projections via the external capsule to superior frontal cortex | -9.50E-04 (-1.75E-03 to -1.46E-04) | 2.05E-02 | 4.98E-02 |
| Right Corticostriate projections via the external capsule to superior parietal cortex | -1.23E-03 (-2.24E-03 to -2.17E-04) | 1.73E-02 | 4.24E-02 |
| Left Corticostriate projections via the external capsule to superior parietal cortex | -1.25E-03 (-2.18E-03 to -3.16E-04) | 8.67E-03 | 2.29E-02 |
| Right Corticostriate projections to inferior frontal cortex | -7.08E-04 (-1.39E-03 to -2.33E-05) | 4.27E-02 | 9.60E-02 |
| Right Corticocortical projections from inferior frontal cortex to superior frontal cortex | -1.30E-03 (-2.01E-03 to -5.81E-04) | 3.83E-04 | 1.23E-03 |
| Left Corticocortical projections from inferior frontal cortex to superior frontal cortex | -1.36E-03 (-2.08E-03 to -6.43E-04) | 2.03E-04 | 6.78E-04 |

Both the original and the false-discovery-rate-corrected p-values for paired t-tests are provided. Only analyses with significant uncorrected p-values are included. values are shown.

**Supplementary Table 15**. Mixed linear model analysis of the association of the NIH Toolbox Total Cognitive Function Composite Score with neurite density (ND) values at baseline after controlling for subjects’ age at time of imaging, sex, BMI z-score at time of imaging, handedness, race, and cranial volume at time of imaging as covariates and adjusting for the highest parental level of education and combined parental income level as random effects.

| **White matter tract** | **Coefficient (95% confidence interval)** | **Original p-value** | **Corrected p-value** |
| --- | --- | --- | --- |
| Left Fornix | 8.88E-04 (9.71E-06 to 1.77E-03) | 4.75E-02 | 1.08E-01 |
| Right Anterior Thalamic Radiations | 1.15E-03 (3.75E-04 to 1.93E-03) | 3.67E-03 | 1.01E-02 |
| Forceps Minor | -2.27E-03 (-3.27E-03 to -1.26E-03) | 9.81E-06 | 3.39E-05 |
| Corpus Callosum | -7.08E-04 (-1.41E-03 to -6.47E-06) | 4.79E-02 | 1.09E-01 |
| Left Parietal Superior Longitudinal Fasiculus | 7.96E-04 (3.54E-05 to 1.56E-03) | 4.03E-02 | 9.31E-02 |
| Left Corticostriate projections via the external capsule to superior parietal cortex | 6.58E-04 (5.60E-06 to 1.31E-03) | 4.81E-02 | 1.09E-01 |

Both the original and the false-discovery-rate-corrected p-values for paired t-tests are provided. Only analyses with significant uncorrected p-values are included. values are shown.

**Supplementary Table 16**. Mixed linear model analysis of the association of the NIH Toolbox Fluid Cognition Composite Score with cortical thickness values at baseline after controlling for subjects’ age at time of imaging, sex, BMI z-score at time of imaging, handedness, race, and cranial volume at time of imaging as covariates and adjusting for the highest parental level of education and combined parental income level as random effects.

| **Region** | **Coefficient (95% confidence interval)** | **Original p-value** | **Corrected p-value** |
| --- | --- | --- | --- |
| Lh-Banks Of Superior Temporal Sulcus | -4.36E-03 (-8.36E-03 to -3.60E-04) | 3.27E-02 | 6.05E-02 |
| Lh-cuneus | -4.97E-03 (-8.45E-03 to -1.50E-03) | 4.98E-03 | 1.06E-02 |
| Lh-entorhinal | 7.17E-03 (3.34E-04 to 1.40E-02) | 3.98E-02 | 7.18E-02 |
| Lh-inferiorparietal | -4.49E-03 (-7.33E-03 to -1.65E-03) | 1.94E-03 | 4.46E-03 |
| Lh-lateraloccipital | -6.44E-03 (-9.48E-03 to -3.41E-03) | 3.12E-05 | 8.88E-05 |
| Lh-lateralorbitofrontal | -4.43E-03 (-7.43E-03 to -1.43E-03) | 3.83E-03 | 8.35E-03 |
| Lh-medialorbitofrontal | -5.25E-03 (-8.71E-03 to -1.79E-03) | 2.97E-03 | 6.60E-03 |
| Lh-middletemporal | -3.85E-03 (-7.61E-03 to -7.61E-05) | 4.56E-02 | 8.12E-02 |
| Lh-parsopercularis | -4.51E-03 (-7.57E-03 to -1.44E-03) | 3.91E-03 | 8.50E-03 |
| Lh-parstriangularis | -5.41E-03 (-8.71E-03 to -2.12E-03) | 1.28E-03 | 2.99E-03 |
| Lh-pericalcarine | -3.94E-03 (-7.70E-03 to -1.81E-04) | 4.00E-02 | 7.19E-02 |
| Lh-postcentral | -6.31E-03 (-9.66E-03 to -2.96E-03) | 2.23E-04 | 5.73E-04 |
| Lh-precuneus | -4.45E-03 (-7.10E-03 to -1.81E-03) | 9.44E-04 | 2.26E-03 |
| Lh-rostralanteriorcingulate | -6.28E-03 (-1.10E-02 to -1.57E-03) | 8.92E-03 | 1.84E-02 |
| Lh-rostralmiddlefrontal | -5.20E-03 (-7.98E-03 to -2.43E-03) | 2.38E-04 | 6.09E-04 |
| Lh-superiorfrontal | -5.07E-03 (-8.17E-03 to -1.97E-03) | 1.34E-03 | 3.14E-03 |
| Lh-superiorparietal | -6.26E-03 (-9.03E-03 to -3.49E-03) | 9.41E-06 | 2.81E-05 |
| Lh-supramarginal | -4.15E-03 (-7.44E-03 to -8.64E-04) | 1.33E-02 | 2.64E-02 |
| Lh-frontalpole | -7.74E-03 (-1.40E-02 to -1.45E-03) | 1.59E-02 | 3.12E-02 |
| Rh-cuneus | -6.33E-03 (-9.74E-03 to -2.92E-03) | 2.73E-04 | 6.91E-04 |
| Rh-fusiform | -3.70E-03 (-6.52E-03 to -8.74E-04) | 1.03E-02 | 2.08E-02 |
| Rh-inferiorparietal | -6.27E-03 (-9.12E-03 to -3.43E-03) | 1.50E-05 | 4.37E-05 |
| Rh-isthmuscingulate | 5.62E-03 (1.86E-03 to 9.38E-03) | 3.38E-03 | 7.44E-03 |
| Rh-lateraloccipital | -7.09E-03 (-1.02E-02 to -4.01E-03) | 6.24E-06 | 1.88E-05 |
| Rh-lingual | -4.16E-03 (-7.20E-03 to -1.12E-03) | 7.29E-03 | 1.53E-02 |
| Rh-medialorbitofrontal | -4.37E-03 (-7.57E-03 to -1.17E-03) | 7.48E-03 | 1.56E-02 |
| Rh-middletemporal | -3.79E-03 (-7.45E-03 to -1.31E-04) | 4.23E-02 | 7.56E-02 |
| Rh-parsopercularis | -3.38E-03 (-6.63E-03 to -1.36E-04) | 4.11E-02 | 7.36E-02 |
| Rh-parstriangularis | -4.08E-03 (-7.29E-03 to -8.73E-04) | 1.27E-02 | 2.52E-02 |
| Rh-postcentral | -5.36E-03 (-9.06E-03 to -1.66E-03) | 4.55E-03 | 9.81E-03 |
| Rh-precuneus | -4.24E-03 (-6.80E-03 to -1.67E-03) | 1.21E-03 | 2.86E-03 |
| Rh-rostralanteriorcingulate | -5.44E-03 (-1.02E-02 to -6.82E-04) | 2.50E-02 | 4.73E-02 |
| Rh-rostralmiddlefrontal | -5.08E-03 (-7.93E-03 to -2.24E-03) | 4.57E-04 | 1.13E-03 |
| Rh-superiorparietal | -6.03E-03 (-8.83E-03 to -3.23E-03) | 2.44E-05 | 7.03E-05 |
| Rh-superiortemporal | -4.07E-03 (-7.58E-03 to -5.58E-04) | 2.31E-02 | 4.39E-02 |
| Rh-supramarginal | -4.04E-03 (-7.46E-03 to -6.20E-04) | 2.06E-02 | 3.96E-02 |

Both the original and the false-discovery-rate-corrected p-values for paired t-tests are provided. Only analyses with significant uncorrected p-values are included. values are shown.

**Supplementary Table 17**. Mixed linear model analysis of the association of the NIH Toolbox Fluid Cognition Composite Score with cortical surface area values at baseline after controlling for subjects’ age at time of imaging, sex, BMI z-score at time of imaging, handedness, race, and cranial volume at time of imaging as covariates and adjusting for the highest parental level of education and combined parental income level as random effects.

| **Region** | **Coefficient (95% confidence interval)** | **Original p-value** | **Corrected p-value** |
| --- | --- | --- | --- |
| Lh-Banks Of Superior Temporal Sulcus | 6.89E+00 (1.85E+00 to 1.19E+01) | 7.38E-03 | 1.79E-02 |
| Lh-caudalanteriorcingulate | 3.94E+00 (6.96E-02 to 7.80E+00) | 4.60E-02 | 9.29E-02 |
| Lh-caudalmiddlefrontal | 2.05E+01 (1.02E+01 to 3.08E+01) | 9.63E-05 | 3.07E-04 |
| Lh-cuneus | 6.26E+00 (6.46E-01 to 1.19E+01) | 2.88E-02 | 6.15E-02 |
| Lh-entorhinal | 3.26E+00 (7.73E-01 to 5.74E+00) | 1.02E-02 | 2.40E-02 |
| Lh-fusiform | 1.49E+01 (6.42E+00 to 2.34E+01) | 5.66E-04 | 1.68E-03 |
| Lh-inferiortemporal | 1.18E+01 (4.71E-01 to 2.31E+01) | 4.12E-02 | 8.49E-02 |
| Lh-lateralorbitofrontal | 1.11E+01 (4.81E+00 to 1.74E+01) | 5.50E-04 | 1.64E-03 |
| Lh-medialorbitofrontal | 9.07E+00 (3.70E+00 to 1.44E+01) | 9.27E-04 | 2.67E-03 |
| Lh-pericalcarine | 6.91E+00 (5.23E-01 to 1.33E+01) | 3.40E-02 | 7.10E-02 |
| Lh-postcentral | 1.60E+01 (3.60E+00 to 2.84E+01) | 1.14E-02 | 2.67E-02 |
| Lh-precentral | 2.38E+01 (1.08E+01 to 3.68E+01) | 3.44E-04 | 1.05E-03 |
| Lh-precuneus | 1.61E+01 (4.63E+00 to 2.75E+01) | 5.89E-03 | 1.48E-02 |
| Lh-rostralanteriorcingulate | 5.98E+00 (2.26E+00 to 9.70E+00) | 1.63E-03 | 4.49E-03 |
| Lh-superiorfrontal | 4.55E+01 (2.46E+01 to 6.64E+01) | 1.98E-05 | 6.72E-05 |
| Lh-superiorparietal | 2.53E+01 (6.44E+00 to 4.42E+01) | 8.59E-03 | 2.05E-02 |
| Lh-superiortemporal | 2.06E+01 (8.62E+00 to 3.27E+01) | 7.65E-04 | 2.24E-03 |
| Lh-supramarginal | 2.78E+01 (9.31E+00 to 4.63E+01) | 3.21E-03 | 8.48E-03 |
| Lh-insula | 9.78E+00 (4.05E+00 to 1.55E+01) | 8.29E-04 | 2.41E-03 |
| Rh-caudalmiddlefrontal | 1.73E+01 (6.57E+00 to 2.80E+01) | 1.57E-03 | 4.34E-03 |
| Rh-fusiform | 8.24E+00 (3.26E-01 to 1.62E+01) | 4.13E-02 | 8.49E-02 |
| Rh-inferiorparietal | 2.71E+01 (7.03E+00 to 4.73E+01) | 8.17E-03 | 1.96E-02 |
| Rh-inferiortemporal | 1.19E+01 (1.88E+00 to 2.20E+01) | 2.00E-02 | 4.42E-02 |
| Rh-lateralorbitofrontal | 1.95E+01 (1.17E+01 to 2.73E+01) | 9.89E-07 | 3.66E-06 |
| Rh-medialorbitofrontal | 1.21E+01 (7.08E+00 to 1.71E+01) | 2.32E-06 | 8.40E-06 |
| Rh-paracentral | 7.77E+00 (2.58E+00 to 1.30E+01) | 3.34E-03 | 8.77E-03 |
| Rh-postcentral | 1.89E+01 (6.16E+00 to 3.17E+01) | 3.67E-03 | 9.58E-03 |
| Rh-precentral | 1.66E+01 (3.00E+00 to 3.01E+01) | 1.67E-02 | 3.75E-02 |
| Rh-precuneus | 1.80E+01 (6.12E+00 to 2.99E+01) | 2.99E-03 | 7.94E-03 |
| Rh-rostralanteriorcingulate | 4.57E+00 (1.62E+00 to 7.52E+00) | 2.38E-03 | 6.38E-03 |
| Rh-rostralmiddlefrontal | 2.21E+01 (1.68E+00 to 4.25E+01) | 3.39E-02 | 7.10E-02 |
| Rh-superiorfrontal | 3.59E+01 (1.46E+01 to 5.73E+01) | 9.74E-04 | 2.79E-03 |
| Rh-superiortemporal | 1.88E+01 (8.52E+00 to 2.90E+01) | 3.34E-04 | 1.02E-03 |
| Rh-supramarginal | 1.84E+01 (3.12E+00 to 3.37E+01) | 1.83E-02 | 4.09E-02 |
| Rh-transversetemporal | 1.40E+00 (2.26E-01 to 2.58E+00) | 1.95E-02 | 4.31E-02 |
| Rh-insula | 8.92E+00 (3.34E+00 to 1.45E+01) | 1.73E-03 | 4.72E-03 |

Both the original and the false-discovery-rate-corrected p-values for paired t-tests are provided. Only analyses with significant uncorrected p-values are included. values are shown.

**Supplementary Table 18**. Mixed linear model analysis of the association of the NIH Toolbox Crystallized Cognition Composite Score with cortical thickness values at baseline after controlling for subjects’ age at time of imaging, sex, BMI z-score at time of imaging, handedness, race, and cranial volume at time of imaging as covariates and adjusting for the highest parental level of education and combined parental income level as random effects.

| **Region** | **Coefficient (95% confidence interval)** | **Original p-value** | **Corrected p-value** |
| --- | --- | --- | --- |
| Lh-parahippocampal | 1.11E-02 (4.74E-03 to 1.76E-02) | 6.49E-04 | 1.59E-03 |
| Lh-paracentral | -4.48E-03 (-8.02E-03 to -9.34E-04) | 1.33E-02 | 2.71E-02 |
| Lh-superiorfrontal | -4.36E-03 (-7.51E-03 to -1.21E-03) | 6.63E-03 | 1.42E-02 |
| Rh-caudalanteriorcingulate | -6.71E-03 (-1.14E-02 to -2.01E-03) | 5.13E-03 | 1.12E-02 |
| Rh-paracentral | -4.04E-03 (-7.37E-03 to -7.14E-04) | 1.73E-02 | 3.47E-02 |
| Rh-parsorbitalis | 5.24E-03 (8.36E-04 to 9.64E-03) | 1.97E-02 | 3.91E-02 |
| Rh-superiorparietal | -3.03E-03 (-5.88E-03 to -1.81E-04) | 3.71E-02 | 7.04E-02 |
| Rh-transversetemporal | 7.15E-03 (2.34E-03 to 1.20E-02) | 3.54E-03 | 7.97E-03 |

Both the original and the false-discovery-rate-corrected p-values for paired t-tests are provided. Only analyses with significant uncorrected p-values are included. values are shown.

**Supplementary Table 19**. Mixed linear model analysis of the association of the NIH Toolbox Crystallized Cognition Composite Score with cortical surface area values at baseline after controlling for subjects’ age at time of imaging, sex, BMI z-score at time of imaging, handedness, race, and cranial volume at time of imaging as covariates and adjusting for the highest parental level of education and combined parental income level as random effects.

| **Region** | **Coefficient (95% confidence interval)** | **Original p-value** | **Corrected p-value** |
| --- | --- | --- | --- |
| Lh-Banks Of Superior Temporal Sulcus | 8.30E+00 (3.17E+00 to 1.34E+01) | 1.51E-03 | 4.20E-03 |
| Lh-caudalanteriorcingulate | 4.39E+00 (4.63E-01 to 8.32E+00) | 2.84E-02 | 6.24E-02 |
| Lh-caudalmiddlefrontal | 1.52E+01 (4.85E+00 to 2.56E+01) | 4.04E-03 | 1.07E-02 |
| Lh-entorhinal | 4.98E+00 (2.46E+00 to 7.50E+00) | 1.05E-04 | 3.41E-04 |
| Lh-fusiform | 1.43E+01 (5.68E+00 to 2.29E+01) | 1.16E-03 | 3.36E-03 |
| Lh-inferiorparietal | 2.74E+01 (9.27E+00 to 4.54E+01) | 3.03E-03 | 8.18E-03 |
| Lh-inferiortemporal | 1.49E+01 (3.40E+00 to 2.65E+01) | 1.11E-02 | 2.68E-02 |
| Lh-medialorbitofrontal | 8.97E+00 (3.51E+00 to 1.44E+01) | 1.28E-03 | 3.65E-03 |
| Lh-postcentral | 2.15E+01 (8.87E+00 to 3.41E+01) | 8.44E-04 | 2.49E-03 |
| Lh-precentral | 2.38E+01 (1.06E+01 to 3.71E+01) | 4.14E-04 | 1.27E-03 |
| Lh-rostralanteriorcingulate | 5.20E+00 (1.42E+00 to 8.98E+00) | 6.95E-03 | 1.76E-02 |
| Lh-superiorparietal | 2.11E+01 (1.83E+00 to 4.03E+01) | 3.18E-02 | 6.90E-02 |
| Lh-superiortemporal | 2.29E+01 (1.07E+01 to 3.52E+01) | 2.47E-04 | 7.79E-04 |
| Lh-supramarginal | 1.89E+01 (2.30E-02 to 3.77E+01) | 4.97E-02 | 1.03E-01 |
| Lh-transversetemporal | 2.84E+00 (9.72E-01 to 4.70E+00) | 2.86E-03 | 7.77E-03 |
| Rh-Banks Of Superior Temporal Sulcus | 6.79E+00 (2.83E+00 to 1.08E+01) | 7.91E-04 | 2.34E-03 |
| Rh-caudalmiddlefrontal | 1.36E+01 (2.75E+00 to 2.45E+01) | 1.41E-02 | 3.31E-02 |
| Rh-entorhinal | 2.88E+00 (6.81E-01 to 5.09E+00) | 1.03E-02 | 2.48E-02 |
| Rh-fusiform | 1.31E+01 (5.06E+00 to 2.12E+01) | 1.43E-03 | 4.01E-03 |
| Rh-inferiorparietal | 2.80E+01 (7.51E+00 to 4.85E+01) | 7.40E-03 | 1.85E-02 |
| Rh-inferiortemporal | 2.07E+01 (1.05E+01 to 3.10E+01) | 7.06E-05 | 2.33E-04 |
| Rh-lateralorbitofrontal | 1.40E+01 (6.04E+00 to 2.20E+01) | 5.66E-04 | 1.71E-03 |
| Rh-medialorbitofrontal | 7.74E+00 (2.64E+00 to 1.28E+01) | 2.96E-03 | 8.00E-03 |
| Rh-middletemporal | 1.53E+01 (4.27E+00 to 2.63E+01) | 6.55E-03 | 1.66E-02 |
| Rh-rostralmiddlefrontal | 2.53E+01 (4.57E+00 to 4.61E+01) | 1.68E-02 | 3.87E-02 |
| Rh-superiorfrontal | 2.49E+01 (3.21E+00 to 4.67E+01) | 2.45E-02 | 5.44E-02 |
| Rh-superiortemporal | 1.62E+01 (5.73E+00 to 2.66E+01) | 2.41E-03 | 6.56E-03 |
| Rh-supramarginal | 2.09E+01 (5.37E+00 to 3.65E+01) | 8.40E-03 | 2.06E-02 |

Both the original and the false-discovery-rate-corrected p-values for paired t-tests are provided. Only analyses with significant uncorrected p-values are included. values are shown.

**Supplementary Table 20**. Mixed linear model analysis of the association of the NIH Toolbox Total Cognitive Function Composite Score with cortical thickness values at baseline after controlling for subjects’ age at time of imaging, sex, BMI z-score at time of imaging, handedness, race, and cranial volume at time of imaging as covariates and adjusting for the highest parental level of education and combined parental income level as random effects.

| **Region** | **Coefficient (95% confidence interval)** | **Original p-value** | **Corrected p-value** |
| --- | --- | --- | --- |
| Lh-cuneus | -4.29E-03 (-7.83E-03 to -7.49E-04) | 1.76E-02 | 3.69E-02 |
| Lh-inferiorparietal | -3.41E-03 (-6.30E-03 to -5.16E-04) | 2.09E-02 | 4.34E-02 |
| Lh-lateraloccipital | -5.66E-03 (-8.75E-03 to -2.56E-03) | 3.37E-04 | 9.41E-04 |
| Lh-lateralorbitofrontal | -4.24E-03 (-7.30E-03 to -1.18E-03) | 6.67E-03 | 1.51E-02 |
| Lh-medialorbitofrontal | -4.55E-03 (-8.08E-03 to -1.03E-03) | 1.14E-02 | 2.47E-02 |
| Lh-middletemporal | -4.02E-03 (-7.86E-03 to -1.75E-04) | 4.04E-02 | 7.64E-02 |
| Lh-parahippocampal | 9.66E-03 (3.27E-03 to 1.61E-02) | 3.03E-03 | 7.26E-03 |
| Lh-parsopercularis | -5.02E-03 (-8.14E-03 to -1.90E-03) | 1.61E-03 | 4.01E-03 |
| Lh-parstriangularis | -4.34E-03 (-7.70E-03 to -9.79E-04) | 1.14E-02 | 2.47E-02 |
| Lh-pericalcarine | -4.22E-03 (-8.05E-03 to -3.84E-04) | 3.11E-02 | 6.09E-02 |
| Lh-postcentral | -5.21E-03 (-8.63E-03 to -1.79E-03) | 2.82E-03 | 6.79E-03 |
| Lh-precuneus | -4.37E-03 (-7.06E-03 to -1.68E-03) | 1.45E-03 | 3.66E-03 |
| Lh-rostralanteriorcingulate | -5.99E-03 (-1.08E-02 to -1.20E-03) | 1.43E-02 | 3.05E-02 |
| Lh-rostralmiddlefrontal | -4.47E-03 (-7.30E-03 to -1.64E-03) | 1.95E-03 | 4.84E-03 |
| Lh-superiorfrontal | -6.28E-03 (-9.44E-03 to -3.12E-03) | 9.65E-05 | 2.85E-04 |
| Lh-superiorparietal | -4.69E-03 (-7.51E-03 to -1.86E-03) | 1.14E-03 | 2.94E-03 |
| Lh-supramarginal | -3.92E-03 (-7.27E-03 to -5.70E-04) | 2.18E-02 | 4.50E-02 |
| Rh-caudalanteriorcingulate | -6.96E-03 (-1.17E-02 to -2.24E-03) | 3.86E-03 | 9.12E-03 |
| Rh-cuneus | -5.01E-03 (-8.49E-03 to -1.53E-03) | 4.75E-03 | 1.11E-02 |
| Rh-fusiform | -2.98E-03 (-5.86E-03 to -1.06E-04) | 4.21E-02 | 7.91E-02 |
| Rh-inferiorparietal | -4.92E-03 (-7.81E-03 to -2.02E-03) | 8.78E-04 | 2.30E-03 |
| Rh-isthmuscingulate | 4.41E-03 (5.78E-04 to 8.25E-03) | 2.41E-02 | 4.88E-02 |
| Rh-lateraloccipital | -6.42E-03 (-9.55E-03 to -3.28E-03) | 6.00E-05 | 1.82E-04 |
| Rh-lateralorbitofrontal | -3.41E-03 (-6.64E-03 to -1.69E-04) | 3.92E-02 | 7.46E-02 |
| Rh-medialorbitofrontal | -3.53E-03 (-6.80E-03 to -2.70E-04) | 3.38E-02 | 6.56E-02 |
| Rh-paracentral | -3.88E-03 (-7.22E-03 to -5.35E-04) | 2.30E-02 | 4.70E-02 |
| Rh-postcentral | -4.14E-03 (-7.92E-03 to -3.66E-04) | 3.16E-02 | 6.17E-02 |
| Rh-precuneus | -3.99E-03 (-6.61E-03 to -1.37E-03) | 2.82E-03 | 6.79E-03 |
| Rh-rostralanteriorcingulate | -6.08E-03 (-1.09E-02 to -1.24E-03) | 1.39E-02 | 2.98E-02 |
| Rh-rostralmiddlefrontal | -3.15E-03 (-6.05E-03 to -2.50E-04) | 3.33E-02 | 6.48E-02 |
| Rh-superiorparietal | -5.40E-03 (-8.24E-03 to -2.56E-03) | 1.92E-04 | 5.48E-04 |

Both the original and the false-discovery-rate-corrected p-values for paired t-tests are provided. Only analyses with significant uncorrected p-values are included. values are shown.

**Supplementary Table 21**. Mixed linear model analysis of the association of the NIH Toolbox Total Cognitive Function Composite Score with cortical surface area values at baseline after controlling for subjects’ age at time of imaging, sex, BMI z-score at time of imaging, handedness, race, and cranial volume at time of imaging as covariates and adjusting for the highest parental level of education and combined parental income level as random effects.

| **Region** | **Coefficient (95% confidence interval)** | **Original p-value** | **Corrected p-value** |
| --- | --- | --- | --- |
| Lh-Banks Of Superior Temporal Sulcus | 9.18E+00 (4.05E+00 to 1.43E+01) | 4.57E-04 | 1.47E-03 |
| Lh-caudalanteriorcingulate | 4.67E+00 (7.33E-01 to 8.60E+00) | 2.01E-02 | 4.67E-02 |
| Lh-caudalmiddlefrontal | 2.04E+01 (9.88E+00 to 3.09E+01) | 1.45E-04 | 4.93E-04 |
| Lh-entorhinal | 5.01E+00 (2.48E+00 to 7.54E+00) | 1.03E-04 | 3.60E-04 |
| Lh-fusiform | 1.69E+01 (8.32E+00 to 2.55E+01) | 1.14E-04 | 3.93E-04 |
| Lh-inferiorparietal | 2.61E+01 (8.07E+00 to 4.41E+01) | 4.55E-03 | 1.25E-02 |
| Lh-inferiortemporal | 1.59E+01 (4.38E+00 to 2.74E+01) | 6.85E-03 | 1.78E-02 |
| Lh-lateralorbitofrontal | 8.03E+00 (1.61E+00 to 1.45E+01) | 1.43E-02 | 3.47E-02 |
| Lh-medialorbitofrontal | 1.12E+01 (5.76E+00 to 1.67E+01) | 5.67E-05 | 2.09E-04 |
| Lh-paracentral | 5.05E+00 (6.08E-01 to 9.50E+00) | 2.59E-02 | 5.88E-02 |
| Lh-postcentral | 2.25E+01 (9.89E+00 to 3.51E+01) | 4.74E-04 | 1.52E-03 |
| Lh-precentral | 2.76E+01 (1.43E+01 to 4.08E+01) | 4.58E-05 | 1.70E-04 |
| Lh-precuneus | 1.50E+01 (3.37E+00 to 2.67E+01) | 1.15E-02 | 2.85E-02 |
| Lh-rostralanteriorcingulate | 6.71E+00 (2.93E+00 to 1.05E+01) | 5.10E-04 | 1.63E-03 |
| Lh-superiorfrontal | 4.00E+01 (1.87E+01 to 6.14E+01) | 2.34E-04 | 7.74E-04 |
| Lh-superiorparietal | 2.78E+01 (8.54E+00 to 4.70E+01) | 4.64E-03 | 1.27E-02 |
| Lh-superiortemporal | 2.55E+01 (1.33E+01 to 3.77E+01) | 4.29E-05 | 1.60E-04 |
| Lh-supramarginal | 2.77E+01 (8.90E+00 to 4.66E+01) | 3.91E-03 | 1.09E-02 |
| Lh-temporalpole | 1.63E+00 (4.95E-02 to 3.21E+00) | 4.33E-02 | 9.21E-02 |
| Lh-transversetemporal | 2.30E+00 (4.26E-01 to 4.17E+00) | 1.62E-02 | 3.84E-02 |
| Lh-insula | 8.09E+00 (2.24E+00 to 1.39E+01) | 6.70E-03 | 1.75E-02 |
| Rh-Banks Of Superior Temporal Sulcus | 4.85E+00 (8.98E-01 to 8.81E+00) | 1.62E-02 | 3.84E-02 |
| Rh-caudalmiddlefrontal | 1.74E+01 (6.49E+00 to 2.83E+01) | 1.77E-03 | 5.21E-03 |
| Rh-entorhinal | 2.26E+00 (6.01E-02 to 4.46E+00) | 4.41E-02 | 9.34E-02 |
| Rh-fusiform | 1.25E+01 (4.48E+00 to 2.06E+01) | 2.29E-03 | 6.59E-03 |
| Rh-inferiorparietal | 3.44E+01 (1.39E+01 to 5.48E+01) | 1.00E-03 | 3.05E-03 |
| Rh-inferiortemporal | 1.95E+01 (9.27E+00 to 2.97E+01) | 1.87E-04 | 6.31E-04 |
| Rh-lateralorbitofrontal | 2.08E+01 (1.29E+01 to 2.88E+01) | 3.02E-07 | 1.25E-06 |
| Rh-medialorbitofrontal | 1.21E+01 (6.95E+00 to 1.72E+01) | 3.75E-06 | 1.49E-05 |
| Rh-middletemporal | 1.26E+01 (1.59E+00 to 2.37E+01) | 2.49E-02 | 5.69E-02 |
| Rh-paracentral | 6.68E+00 (1.39E+00 to 1.20E+01) | 1.33E-02 | 3.24E-02 |
| Rh-postcentral | 1.90E+01 (5.99E+00 to 3.20E+01) | 4.21E-03 | 1.17E-02 |
| Rh-precuneus | 1.84E+01 (6.31E+00 to 3.05E+01) | 2.87E-03 | 8.18E-03 |
| Rh-rostralanteriorcingulate | 3.88E+00 (8.78E-01 to 6.87E+00) | 1.13E-02 | 2.81E-02 |
| Rh-rostralmiddlefrontal | 2.66E+01 (5.88E+00 to 4.73E+01) | 1.19E-02 | 2.93E-02 |
| Rh-superiorfrontal | 3.38E+01 (1.21E+01 to 5.55E+01) | 2.23E-03 | 6.45E-03 |
| Rh-superiorparietal | 2.13E+01 (2.51E+00 to 4.02E+01) | 2.63E-02 | 5.94E-02 |
| Rh-superiortemporal | 2.06E+01 (1.02E+01 to 3.11E+01) | 1.07E-04 | 3.73E-04 |
| Rh-supramarginal | 2.44E+01 (8.81E+00 to 4.00E+01) | 2.15E-03 | 6.25E-03 |
| Rh-transversetemporal | 1.30E+00 (1.02E-01 to 2.50E+00) | 3.34E-02 | 7.35E-02 |
| Rh-insula | 6.29E+00 (6.02E-01 to 1.20E+01) | 3.02E-02 | 6.69E-02 |

Both the original and the false-discovery-rate-corrected p-values for paired t-tests are provided. Only analyses with significant uncorrected p-values are included. values are shown.

**Supplementary Table 22**. Mixed linear model analysis of the association of the NIH Toolbox Fluid Cognition Composite Score with fMRI functional connectivity values at baseline after controlling for subjects’ age at time of imaging, sex, BMI z-score at time of imaging, handedness, race, and cranial volume at time of imaging as covariates and adjusting for the highest parental level of education and combined parental income level as random effects.

| **Connectivity** | **Coefficient (95% confidence interval)** | **Original**  **p-value** | **Corrected p-value** |
| --- | --- | --- | --- |
| Auditory Network And Cingulo-parietal Network | -2.93E-03 (-4.80E-03 to -1.06E-03) | 2.12E-03 | 7.06E-03 |
| Auditory Network And Dorsal Attention Network | -1.51E-03 (-2.68E-03 to -3.48E-04) | 1.09E-02 | 3.17E-02 |
| Auditory Network And Retrosplenial Temporal Network | -2.46E-03 (-4.28E-03 to -6.38E-04) | 8.12E-03 | 2.42E-02 |
| Auditory Network And Ventral Attention Network | 1.54E-03 (9.75E-05 to 2.99E-03) | 3.64E-02 | 9.01E-02 |
| Cingulo-opercular Network And Cingulo-parietal Network | -2.15E-03 (-4.13E-03 to -1.73E-04) | 3.31E-02 | 8.27E-02 |
| Cingulo-opercular Network And Fronto-parietal Network | -1.88E-03 (-3.12E-03 to -6.42E-04) | 2.91E-03 | 9.51E-03 |
| Cingulo-opercular Network And Retrosplenial Temporal Network | 2.08E-03 (2.30E-04 to 3.93E-03) | 2.75E-02 | 7.04E-02 |
| Cingulo-opercular Network And Salience Network | -2.27E-03 (-4.07E-03 to -4.80E-04) | 1.29E-02 | 3.71E-02 |
| Cingulo-opercular Network And Visual Network | 2.83E-03 (1.21E-03 to 4.46E-03) | 6.41E-04 | 2.36E-03 |
| Cingulo-parietal Network And Cingulo-parietal Network | 8.49E-03 (3.32E-03 to 1.37E-02) | 1.29E-03 | 4.40E-03 |
| Cingulo-parietal Network And Dorsal Attention Network | -2.25E-03 (-4.37E-03 to -1.38E-04) | 3.68E-02 | 9.06E-02 |
| Cingulo-parietal Network And Fronto-parietal Network | -2.31E-03 (-4.10E-03 to -5.15E-04) | 1.16E-02 | 3.36E-02 |
| Cingulo-parietal Network And Retrosplenial Temporal Network | 6.66E-03 (3.67E-03 to 9.64E-03) | 1.25E-05 | 6.29E-05 |
| Cingulo-parietal Network And Salience Network | -4.77E-03 (-7.38E-03 to -2.16E-03) | 3.35E-04 | 1.31E-03 |
| Cingulo-parietal Network And Sensorimotor Hand Network | -2.70E-03 (-4.60E-03 to -8.07E-04) | 5.21E-03 | 1.62E-02 |
| Cingulo-parietal Network And Ventral Attention Network | -2.70E-03 (-4.49E-03 to -9.09E-04) | 3.12E-03 | 1.01E-02 |
| Default Network And Fronto-parietal Network | -2.19E-03 (-3.35E-03 to -1.02E-03) | 2.34E-04 | 9.39E-04 |
| Default Network And Retrosplenial Temporal Network | -2.11E-03 (-3.78E-03 to -4.34E-04) | 1.36E-02 | 3.88E-02 |
| Default Network And Ventral Attention Network | -1.41E-03 (-2.75E-03 to -7.60E-05) | 3.83E-02 | 9.41E-02 |
| Dorsal Attention Network And Dorsal Attention Network | 2.31E-03 (4.90E-04 to 4.13E-03) | 1.29E-02 | 3.69E-02 |
| Dorsal Attention Network And Sensorimotor Hand Network | -1.43E-03 (-2.59E-03 to -2.71E-04) | 1.56E-02 | 4.29E-02 |
| Dorsal Attention Network And Sensorimotor Mouth Network | -3.00E-03 (-4.50E-03 to -1.51E-03) | 8.38E-05 | 3.62E-04 |
| Dorsal Attention Network And Visual Network | 1.58E-03 (1.12E-04 to 3.04E-03) | 3.49E-02 | 8.69E-02 |
| Fronto-parietal Network And Fronto-parietal Network | 1.58E-03 (6.35E-05 to 3.10E-03) | 4.11E-02 | 9.96E-02 |
| Fronto-parietal Network And None Network | -6.52E-04 (-1.24E-03 to -6.39E-05) | 2.98E-02 | 7.53E-02 |
| Fronto-parietal Network And Salience Network | -1.67E-03 (-3.33E-03 to -2.32E-05) | 4.69E-02 | 1.12E-01 |
| Fronto-parietal Network And Sensorimotor Hand Network | -1.28E-03 (-2.45E-03 to -1.08E-04) | 3.23E-02 | 8.09E-02 |
| Fronto-parietal Network And Visual Network | 1.52E-03 (2.43E-04 to 2.80E-03) | 1.97E-02 | 5.31E-02 |
| None Network And Retrosplenial Temporal Network | -1.52E-03 (-2.45E-03 to -5.94E-04) | 1.30E-03 | 4.42E-03 |
| Retrosplenial Temporal Network And Ventral Attention Network | -2.11E-03 (-3.83E-03 to -3.83E-04) | 1.66E-02 | 4.54E-02 |
| Salience Network And Salience Network | -4.49E-03 (-7.70E-03 to -1.28E-03) | 6.15E-03 | 1.88E-02 |
| Sensorimotor Hand Network And Ventral Attention Network | 2.63E-03 (1.40E-03 to 3.86E-03) | 2.76E-05 | 1.30E-04 |
| Ventral Attention Network And Ventral Attention Network | -2.09E-03 (-3.75E-03 to -4.30E-04) | 1.36E-02 | 3.88E-02 |

Both the original and the false-discovery-rate-corrected p-values for paired t-tests are provided. Only analyses with significant uncorrected p-values are included. values are shown.

**Supplementary Table 23**. Mixed linear model analysis of the association of the NIH Toolbox Crystallized Cognition Composite Score with fMRI functional connectivity values at baseline after controlling for subjects’ age at time of imaging, sex, BMI z-score at time of imaging, handedness, race, and cranial volume at time of imaging as covariates and adjusting for the highest parental level of education and combined parental income level as random effects.

| **Connectivity** | **Coefficient (95% confidence interval)** | **Original**  **p-value** | **Corrected p-value** |
| --- | --- | --- | --- |
| Auditory Network And Retrosplenial Temporal Network | -2.12E-03 (-3.96E-03 to -2.72E-04) | 2.45E-02 | 6.59E-02 |
| Cingulo-opercular Network And Fronto-parietal Network | -1.51E-03 (-2.77E-03 to -2.46E-04) | 1.92E-02 | 5.39E-02 |
| Cingulo-opercular Network And Ventral Attention Network | -2.03E-03 (-3.53E-03 to -5.38E-04) | 7.70E-03 | 2.38E-02 |
| Cingulo-opercular Network And Visual Network | 2.87E-03 (1.22E-03 to 4.52E-03) | 6.52E-04 | 2.43E-03 |
| Cingulo-parietal Network And Cingulo-parietal Network | 7.23E-03 (1.34E-03 to 1.31E-02) | 1.61E-02 | 4.63E-02 |
| Cingulo-parietal Network And Retrosplenial Temporal Network | 3.96E-03 (9.16E-04 to 7.00E-03) | 1.08E-02 | 3.23E-02 |
| Cingulo-parietal Network And Salience Network | -3.19E-03 (-5.84E-03 to -5.40E-04) | 1.83E-02 | 5.18E-02 |
| Default Network And Retrosplenial Temporal Network | -3.01E-03 (-4.71E-03 to -1.31E-03) | 5.25E-04 | 2.01E-03 |
| Default Network And Sensorimotor Hand Network | -1.74E-03 (-2.95E-03 to -5.29E-04) | 4.85E-03 | 1.56E-02 |
| Fronto-parietal Network And None Network | -9.90E-04 (-1.59E-03 to -3.93E-04) | 1.15E-03 | 4.03E-03 |
| Fronto-parietal Network And Retrosplenial Temporal Network | 2.82E-03 (1.31E-03 to 4.34E-03) | 2.57E-04 | 1.03E-03 |
| None Network And None Network | -7.59E-04 (-1.33E-03 to -1.85E-04) | 9.56E-03 | 2.91E-02 |
| Retrosplenial Temporal Network And Sensorimotor Hand Network | -1.53E-03 (-3.03E-03 to -3.44E-05) | 4.50E-02 | 1.11E-01 |
| Salience Network And Salience Network | -4.26E-03 (-7.52E-03 to -1.00E-03) | 1.04E-02 | 3.13E-02 |

Both the original and the false-discovery-rate-corrected p-values for paired t-tests are provided. Only analyses with significant uncorrected p-values are included. values are shown.

**Supplementary Table 24**. Mixed linear model analysis of the association of the NIH Toolbox Total Cognitive Function Composite Score with fMRI functional connectivity values at baseline after controlling for subjects’ age at time of imaging, sex, BMI z-score at time of imaging, handedness, race, and cranial volume at time of imaging as covariates and adjusting for the highest parental level of education and combined parental income level as random effects.

| **Connectivity** | **Coefficient (95% confidence interval)** | **Original**  **p-value** | **Corrected p-value** |
| --- | --- | --- | --- |
| Auditory Network And Cingulo-parietal Network | -2.42E-03 (-4.32E-03 to -5.17E-04) | 1.27E-02 | 3.81E-02 |
| Auditory Network And Dorsal Attention Network | -1.40E-03 (-2.59E-03 to -2.15E-04) | 2.06E-02 | 5.72E-02 |
| Auditory Network And Retrosplenial Temporal Network | -2.76E-03 (-4.61E-03 to -9.04E-04) | 3.54E-03 | 1.19E-02 |
| Auditory Network And Ventral Attention Network | 1.48E-03 (1.14E-05 to 2.96E-03) | 4.82E-02 | 1.16E-01 |
| Cingulo-opercular Network And Fronto-parietal Network | -2.17E-03 (-3.43E-03 to -9.06E-04) | 7.57E-04 | 3.01E-03 |
| Cingulo-opercular Network And Retrosplenial Temporal Network | 2.21E-03 (3.25E-04 to 4.09E-03) | 2.15E-02 | 5.95E-02 |
| Cingulo-opercular Network And Salience Network | -2.03E-03 (-3.86E-03 to -2.00E-04) | 2.96E-02 | 7.85E-02 |
| Cingulo-opercular Network And Ventral Attention Network | -1.63E-03 (-3.12E-03 to -1.47E-04) | 3.12E-02 | 8.16E-02 |
| Cingulo-opercular Network And Visual Network | 3.47E-03 (1.81E-03 to 5.12E-03) | 4.03E-05 | 2.15E-04 |
| Cingulo-parietal Network And Cingulo-parietal Network | 9.00E-03 (3.72E-03 to 1.43E-02) | 8.39E-04 | 3.31E-03 |
| Cingulo-parietal Network And Retrosplenial Temporal Network | 6.55E-03 (3.51E-03 to 9.60E-03) | 2.46E-05 | 1.35E-04 |
| Cingulo-parietal Network And Salience Network | -4.73E-03 (-7.39E-03 to -2.07E-03) | 4.83E-04 | 2.05E-03 |
| Cingulo-parietal Network And Ventral Attention Network | -2.42E-03 (-4.25E-03 to -6.01E-04) | 9.17E-03 | 2.89E-02 |
| Default Network And Retrosplenial Temporal Network | -3.00E-03 (-4.71E-03 to -1.30E-03) | 5.52E-04 | 2.29E-03 |
| Dorsal Attention Network And Fronto-parietal Network | 1.52E-03 (3.17E-04 to 2.73E-03) | 1.33E-02 | 3.97E-02 |
| Dorsal Attention Network And Sensorimotor Mouth Network | -2.40E-03 (-3.93E-03 to -8.73E-04) | 2.07E-03 | 7.49E-03 |
| Fronto-parietal Network And None Network | -9.42E-04 (-1.54E-03 to -3.43E-04) | 2.04E-03 | 7.41E-03 |
| Fronto-parietal Network And Retrosplenial Temporal Network | 1.97E-03 (4.49E-04 to 3.49E-03) | 1.11E-02 | 3.41E-02 |
| Fronto-parietal Network And Salience Network | -2.16E-03 (-3.84E-03 to -4.75E-04) | 1.20E-02 | 3.62E-02 |
| Fronto-parietal Network And Visual Network | 1.66E-03 (3.58E-04 to 2.97E-03) | 1.25E-02 | 3.76E-02 |
| None Network And None Network | -7.06E-04 (-1.29E-03 to -1.22E-04) | 1.78E-02 | 5.06E-02 |
| Retrosplenial Temporal Network And Sensorimotor Hand Network | -1.72E-03 (-3.23E-03 to -2.22E-04) | 2.45E-02 | 6.67E-02 |
| Retrosplenial Temporal Network And Ventral Attention Network | -2.07E-03 (-3.83E-03 to -3.17E-04) | 2.07E-02 | 5.74E-02 |
| Salience Network And Salience Network | -5.49E-03 (-8.76E-03 to -2.22E-03) | 1.01E-03 | 3.85E-03 |
| Sensorimotor Hand Network And Ventral Attention Network | 1.91E-03 (6.54E-04 to 3.16E-03) | 2.87E-03 | 1.00E-02 |

Both the original and the false-discovery-rate-corrected p-values for paired t-tests are provided. Only analyses with significant uncorrected p-values are included. values are shown.
